# Multisensory integration in the mouse cortical connectome using a network diffusion model

**DOI:** 10.1162/netn_a_00164

**Published:** 2020-11-01

**Authors:** Kamal Shadi, Eva Dyer, Constantine Dovrolis

**Affiliations:** School of Computer Science, Georgia Institute of Technology, Atlanta, GA, USA; Department of Biomedical Engineering, Georgia Institute of Technology, Atlanta, GA, USA; School of Computer Science, Georgia Institute of Technology, Atlanta, GA, USA

**Keywords:** Mouse connectome, Claustrum, Parietal temporal cortex, Network diffusion cascade, Asynchronous linear threshold model, Hourglass effect

## Abstract

Having a structural network representation of connectivity in the brain is instrumental in analyzing communication dynamics and neural information processing. In this work, we make steps towards understanding multisensory information flow and integration using a network diffusion approach. In particular, we model the flow of evoked activity, initiated by stimuli at primary sensory regions, using the asynchronous linear threshold (ALT) diffusion model. The ALT model captures how evoked activity that originates at a given region of the cortex “ripples through” other brain regions (referred to as an *activation cascade*). We find that a small number of brain regions–the claustrum and the parietal temporal cortex being at the top of the list–are involved in almost all cortical sensory streams. This suggests that the cortex relies on an hourglass architecture to first integrate and compress multisensory information from multiple sensory regions, before utilizing that lower dimensionality representation in higher level association regions and more complex cognitive tasks.

## Author Summary

Having a structural network representation of connectivity in the brain is instrumental in analyzing communication dynamics and neural information processing. In this work, we make steps towards understanding multisensory information flow and integration using a network diffusion approach. In particular, we model the flow of evoked activity, initiated by stimuli at primary sensory regions, using the asynchronous linear threshold (ALT) diffusion model. The ALT model captures how evoked activity that originates at a given region of the cortex “ripples through” other brain regions (referred to as an activation cascade). We apply the ALT model to the mouse connectome provided by the Allen Institute for Brain Science. A first result, using functional datasets based on voltage-sensitive dye (VSD) imaging, is that the ALT model, despite its simplicity, predicts the temporal ordering of each sensory activation cascade quite accurately. We further apply this model to study multisensory integration and find that a small number of brain regionsthe claustrum and the parietal temporal cortex being at the top of the listare involved in almost all cortical sensory streams. This suggests that the cortex relies on an hourglass architecture to first integrate and compress multisensory information from multiple sensory regions, before utilizing that lower dimensionality representation in higher level association regions and more complex cognitive tasks.

## INTRODUCTION

Perception requires the integration of multiple sensory inputs across distributed areas throughout the brain (Stein & Meredith, 1993). While sensory integration at the behavioral level has been extensively studied (Seilheimer, Rosenberg, & Angelaki, 2014), the network and system-level mechanisms underlying multisensory integration (MSI) are still not well understood, especially in terms of the role that cortex plays. The traditional view of primary cortical sensory areas as processing a single modality is rapidly shifting towards a view of the cortex as highly integrated and multisensory (Meredith, 2002). The somatosensory, visual, auditory, gustatory, and other sensory streams come together (integrate) and separate (diverge) to be processed in different parts of the cortex. The neural basis of how these sensory streams are processed and how they generate a coherent perceptual state remains elusive (Shine et al., 2019). It is likely that this state is created and regulated by multiple structures distributed throughout the cortex working together in concert (Ghazanfar & Schroeder, 2006).

To understand the architectural principles that enable MSI, we need data and models that span the entire brain—focusing not on individual neurons, regions, or even circuits, but on distributed networks. The connectome is thus a potentially powerful tool for studying MSI. However, it would not be enough to just know how different brain regions are connected anatomically. Rather, we need models that combine structure (connectomics) with function (Barrat, Barthelemy, & Vespignani, 2008) to address the question of which connections and paths are “activated” by different sensory modalities (Abdelnour, Dayan, Devinsky, Thesen, & Raj, 2018; Abdelnour, Voss, & Raj, 2014; Park & Friston, 2013; Sporns, 2013; Zhang et al., 2017). Having both a structural network and a functional model in hand, we can begin to tackle the problem of discovering the networks that support and constrain MSI.

Here, we adopt a variation of the asynchronous linear threshold (ALT) network diffusion model (Worrell, Rumschlag, Betzel, Sporns, & Mišić, 2017) to capture the communication dynamics of networks that contribute to MSI. In particular, we focus on how information propagates throughout the brain, starting from different primary sensory regions (e.g., primary visual cortex, auditory cortex, and different somatosensory regions). The ALT model assumes that a “node” (brain region)^1^ becomes active when more than a fraction of the neighboring nodes it receives afferent projections from are active. We use a variation of this model with weighted connections, where the weights are based on the connection density of the projections (Worrell et al., 2017). The ALT model is simple, yet it incorporates information about distances between areas (to model connection delays) and uses local information (a thresholding nonlinearity) to potentially gate the flow of information. With such a model, it is possible to understand how activation cascades propagate in the brain, and then combine them to study the global architecture of MSI.

We apply this model to the Mouse Connectivity Atlas provided by the Allen Institute for Brain Science (Oh et al., 2014). This connectome has been available since 2014, and consists of estimates of the connection density between cortical as well as subcortical regions, providing access to information about whole-brain connectivity across functionally and structurally distinct regions. By coupling the ALT network diffusion model with this representation of the connectome, we can ask questions such as the following: What is the relative order in which different regions get activated after, say, a visual or auditory stimulation? Which are the most central regions for each activation cascade? Are these cascades largely independent of each other, or are there a few “bottleneck” regions through which almost all cascades go through? If so, which are these regions and what is their topological role in each cascade?

To examine the accuracy of the ALT model, we use voltage-sensitive dye (VSD) imaging to capture how activity that is triggered from unisensory stimulation in a specific primary sensory area (e.g., primary visual cortex, VisP; primary auditory cortex, AudP) propagates throughout the cortex (Mohajerani et al., 2013). Such comparisons reveal that the ALT model correctly predicts the temporal ordering of node activations in a cascade when the VSD data provide sufficient temporal resolution to permit activation time comparisons. This suggests that despite the simplicity of linear threshold models, they can provide a useful approach for studying communication dynamics in brain networks.

We then aggregate the unisensory activation cascades predicted by the ALT model to investigate the architecture of MSI. To do so, we consider the number of activation paths that traverse each node across all of the different unisensory cascades inferred by the ALT model. We find that a small set of brain regions (around ten) form a “bottleneck” through which almost all such activation paths traverse. The claustrum (CLA), despite its small size, is the most central bottleneck region in the flow of sensory information from primary sources towards higher level brain regions (Mathur, 2014). The posterior parietal (PTLp) cortex is the second bottleneck node, covering almost as many activation paths as CLA.

## METHODS AND DATA

The ALT model focuses on how stimulation of one cortical region propagates to the rest of the brain when constrained by the underlying connectome. In the rest of this section, we describe the main components of our approach: the mouse connectome, the ALT diffusion model, the hourglass network analysis framework, and the VSD-based model validation.

### Structural Network

The structural (i.e., anatomical) network we analyze is the Allen Mouse Brain Connectivity Atlas (Oh et al., 2014), which is based on tracking axonal projections labeled by viral tracers. It consists of 213 regoins of interest (ROIs) that cover the entire brain—all tracer injections, however, were performed at the right hemisphere (Oh et al., 2014). This means that the contralateral connections from the left hemisphere to the right and the ipsilateral connections at the left hemisphere are not mapped. For this reason we analyze only right hemisphere connections.

The strength of the connection from a source ROI to a target ROI is quantified by a metric that Oh et al. (2014) refer to as *connection density*. This metric can be thought of as the total number of axons from the source ROI to the target ROI, normalized by the size of the target ROI.

Each connection is associated with a *p* value that quantifies the statistical confidence that that connection exists (Oh et al., 2014). We filter out connections with *p* value higher than 0.05.^2^ The reason that we do not filter connections based on their weight is because there are many weak but statistically significant connections, as shown in Figure SI-1. It is well known that weak connections can play an important role in network diffusion phenomena as long as there are many of them (Gallos, Makse, & Sigman, 2012).

The physical length of the connections is approximated based on the Euclidean distance between the two corresponding ROIs’ centroids. This is only an approximation but it is probably accurate because the mouse cortical surface is small and smooth with little folding (Mota & Herculano-Houzel, 2012; Sun & Hevner, 2014). Additionally, as shown in Section “Robustness of tau-core nodes,” the results are robust to the selection of the connection weights or physical lengths.

In most of the analysis, we consider a portion of the mouse connectome that consists of the 67 ROIs that cover the four right hemisphere components of the cerebral cortex: isocortex, olfactory areas, hippocampal formation, and cortical subplate. The complete list of these ROIs is given in Table SI-1. We exclude subcortical ROIs that reside in the cerebellar cortex, cerebellar nuclei, striatum, medulla, pallidum, midbrain, pons, thalamus, and hypothalamus for the following reasons.

The cortical network, denoted as *N*_*c*_, consists of 617 directed edges between 67 nodes; each node corresponds to one of the ROIs in our model. The density of *N*_*c*_ is 13.9%. The distribution of edge weights is skewed, with 80% of the edges having a weight of less than 5 and few edges having a weight of up to 40. The distribution of edge lengths is almost uniform in the 1–7 mm range. The diameter of the network (maximum shortest path length between any two nodes) is 7 hops, while a node is on the average about 4 hops away from any other node. The average in-degree of each node is 9.2 connections (*σ* = 3.1), while the out-degree distribution has the same mean but larger variability (*σ* = 8.3). Additionally, the network *N*_*c*_ is strongly clustered, with an average clustering coefficient of 60% (Fagiolo, 2007).

The 10 primary sensory regions associated with the visual, auditory, gustatory, and olfactory systems, as well as 6 somatosensory regions for different body parts, have a special role in our analysis: They are viewed as *sources* of sensory information in the cortex (Bota, Sporns, & Swanson, 2015; Taylor, Hobbs, Burroni, & Siegelmann, 2015). The complete list and location of these ten ROIs in the Allen Mouse Brain Atlas are shown at Table SI-2.

### The ALT Model and Activation Cascades

The connectome is a structural network and so it constraints, but it does not determine by itself, the paths through which information flows in the brain. To study that flow, we also need to model how dynamic brain activity propagates on the connectome.

We choose a simple network diffusion linear threshold model (Granovetter, 1978), mostly because it involves a single parameter—more realistic neural mass models, such as Wilson-Cowan, depend on several parameters (Sanz-Leon, Knock, Spiegler, & Jirsa, 2015). The model assumes that nodes are either *inactive* (state = 0) or *active* (state = 1). In the model’s simplest form, a node *i* becomes active when more than a fraction *θ* of its neighbors become *active*. Here, we deploy a variation for directed and weighted networks in which each edge is associated with a communication delay and a weight, referred to as asynchronous linear thereshold (ALT) model.

Specifically, the state of node *n*_*i*_ is represented by *s*_*i*_(*t*), the neighbors of *n*_*i*_ with an incoming edge to *n*_*i*_ are denoted by *N*_*in*_(*n*_*i*_), the communication delay from node *n*_*j*_ ∈ *N*_*in*_(*n*_*i*_) is *t*_*ji*_, while the weight of that connection is *w*_*ji*_. Initially, the state of every node is set to 0, except the *source* node of the activation cascade, which is set to active at time *t* = 0. The state of each node *n*_*i*_ is then updated asynchronously based on the state of its neighbors as follows:

$$s_{i}(t)=1\;\text{if}\;\underset{j\in N_{in}(i)}{\sum }w_{ji}\;s_{j}(t-t_{ji})\;>\;\theta ,$$ (1)

where *θ* represents the activation threshold.

**Figure 1.**
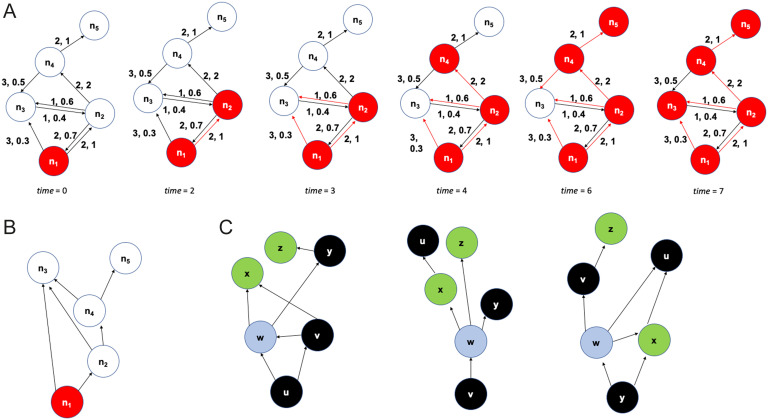
Illustration of ALT model and *τ*-core analysis. (A) A toy example of a five-node network on which we run the ALT model. Each edge is marked with a communication delay, followed by a weight. The activation threshold is *θ* = 1. The black edges represent the underlying structural network, while the red unidirectional edges represent the activation cascade as it unfolds over time. (B) The activation cascade (a directed acyclic graph) for the previous toy example. The source of the cascade is *n*_1_. (C) A different toy example with three activation cascades (the sources are nodes *u*, *v*, and *y*). The total number of source-target paths is 12 (5 at the left, 3 at the middle, and 4 at the right). Node *w* has the highest path centrality (*P*(*w*) = 10/12). If *τ* ≤ 10/12, the *τ*-core consists of only that node.

Figure 1A illustrates the ALT model with a toy example, where we can think of each node as a brain region. The (directed) edges between nodes represent the structural connections between the corresponding regions. In this example, the activation threshold *θ* is set to 1. For this value, the cascade covers the entire network node within 7 time steps. If *θ* was larger than 1, the cascade would not take place—the only active node would be *n*_1_. As we will see in the next section, this sharp transition between not having a cascade and a complete cascade as *θ* decreases also occurs in the mouse connectome.

An activation cascade also reveals the node(s) that contribute in a causal manner in the activation of a node. For example, the activation of *n*_1_ and of *n*_2_ in the previous example is not sufficient to activate *n*_3_; the latter is activated only when *n*_1_, *n*_2_, and *n*_4_ are all active. Suppose that $t_{i}^{a}$ denotes the time at which *n*_*i*_ becomes active according to the ALT model. We say that node *n*_*j*_ contributes in the activation of *n*_*i*_ (denoted by $n_{j}\to n_{i}$) if *n*_*j*_ ∈ *N*_*in*_(*n*_*i*_) and $t_{i}^{a}\geq t_{j}^{a}+t_{ji}$. In other words, the nodes *j* that contribute to the activation of *n*_*i*_ have a connection to *n*_*i*_ and they should be active at least *t*_*ji*_ time units before the activation of *n*_*i*_. The set of relations $n_{j}\to n_{i}$ form a directed acyclic graph (DAG) with a single source node that covers all nodes that participate in the cascade. The DAG edges are a subset of the connections in the underlying structural network. The cascade for the previous example is shown in Figure 1B.

### Analysis of Activation Cascades

An activation cascade consists of a collection of *source-target* paths, with each such path originating at the source of the cascade and terminating at a node without any outgoing edges in the cascade. A source-target path is a sequence of ROI activations that propagate in a causal manner from the source node to a target node. For instance, the cascade of Figure 1B includes three source-target paths from *n*_1_ to *n*_3_ because the activation of the latter requires the activation of *n*_1_, *n*_2_, and *n*_4_.

After constructing a cascade for each source, we use network analysis to identify the nodes that play a more central role in the collection of all cascades. The centrality metric we use has the following graph theoretic interpretation: For each node *v*, the path centrality  *P*(*v*) of node *v* is the fraction of all source-target paths, across all cascades, that traverse *v* (following the terminology of Sabrin & Dovrolis, 2017). Figure 1C illustrates this metric with three small cascades. Nodes with higher path centrality (PC) are expected to be more important because the activation cascades depend more heavily on them. Note that a source node is traversed by all source-target paths in its own cascade but it may have low path centrality when we consider the collection of all cascades.

The PC metric quantifies the importance of each node in isolation. We are interested, on the other hand, in the smallest set of nodes that can jointly cover almost all source-target paths in the given set of cascades. To answer this question, we adopt the τ-core definition of Sabrin and Dovrolis (2017): The *τ*-core is the minimal set of nodes such that the fraction of source-target paths that traverse any node in the set is at least *τ*. The *τ*-core problem is NP-Hard for *τ* < 1 (Sabrin & Dovrolis, 2017). It can be approximated with a greedy heuristic in which the node with the highest PC joins the set in each iteration. That node is then removed from all cascades it appears in. The PC of the remaining nodes is recomputed after each iteration. If the *τ*-core is a small set, relative to the total number of nodes in the network, the nodes of that set can be thought of as the *bottleneck* of the activation cascades (see Figure 1C for an example).

### Comparison of Modeling Results With Functional Data

To examine whether the ALT model can accurately predict the propagation of sensory stimulation in the cortex, we need an experimental setup in which we can stimulate different sensory modalities of a living animal, and monitor at the same time and in a fine temporal resolution (in the order of a millisecond) the activity of different cortical neural populations (in a spatial resolution of few *μ*m).

Such experiments are possible today, relying on technologies such as calcium imaging or voltage-sensitive dies (VSD) in conjuction with fluorescence microscopy. Here, we leverage the experimental results of an earlier study to examine the accuracy of the ALT model in the context of whole-cortex imaging in mice under single sensory stimulation (Mohajerani et al., 2013). In brief, the experiments include five types of sensory stimulation: visual (flash), auditory (tone), whisker touch, forelimb touch, and hindlimb touch. Each stimulation experiment is repeated 10 times and on several different animals (we analyze data for five animals). The recorded images cover almost the entire cortical surface, have a temporal resolution of 6.67 ms, and a size of 128 × 128 pixels at a spatial resolution of 50*μ*m/pixel. As it will become clear in Section “Model validation,” the experimental spatial resolution is sufficient for our purposes, given that each network node in the ALT model refers to an entire cortical ROI of the Allen mouse brain atlas. The temporal resolution, however, is marginally sufficient because for about 20–30% of all ROI pairs we cannot tell which ROI gets activated first as they appear to be activated during the same frame.

To compare the VSD-based results with our modeling results, we perform the following steps:

**Step 1. Register:** Register the native cortical surface of each animal to the Allen Mouse Brain Atlas. This step is performed using an affine transformation that minimizes the least-squares error between the coordinates of the centroid of a primary sensory ROI (e.g., VISp) in the Allen atlas, and the coordinates of the pixel that first gets activated after the corresponding sensory stimulation (visual in this example). We use five primary sensory ROIs to construct this transformation: VISp, SSp-bfd, SSp-ll, SSp-ul, and AUD-p.

**Step 2. Parcellate into ROIs:** Parcellate the native cortical surface into ROIs using the Allen Mouse Brain Atlas and the previous affine transformation. Some cortical ROIs are not visible in the VSD images: FRP, PL, ACAd, VISpl, and VISpor. Also, the MOs region is only partially captured in the VSD images.

**Figure 2.**
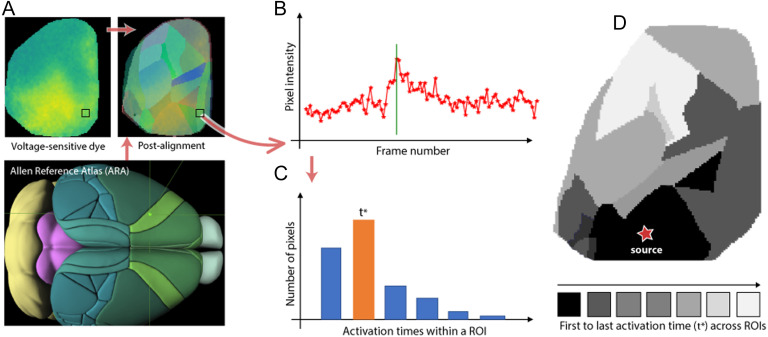
VSD data-processing pipeline. (A) Lower: The Allen Reference Atlas (ARA). Upper left: A sample VSD image covering most of the left cortical surface five frames after visual stimulation. Upper right: The ROIs at the left ARA cortical surface mapped to the native cortical surface of an animal. (B) The activation time of a pixel is defined as the frame of maximum poststimulus VSD signal at that pixel. (C) The activation time of an ROI is defined as the activation time of *most* pixels in that ROI. (D) The output of this pipeline is an activation time for each ROI, depicted here with a gray scale map (black for the first ROI activation and white for the last).

**Step 3. Estimate activation time:** Estimate an *activation time* for each ROI in the experimental data. To perform this step, we first identify the maximum poststimulus activity for each pixel in that ROI—this is defined as the activation time of that pixel. The image frame that corresponds to the activation time for most pixels of that ROI is defined as the activation time of the ROI. This processing pipeline is summarized in Figure 2.

The ALT model, on the other hand, models each cortical ROI as a single node, and it assumes that the transition of each node from inactive to active occurs instantaneously. We examine the consistency of the temporal ordering of activations in the ALT model and in the VSD experiments using both Kendall’s rank correlation coefficient as well as using the following approach: If *X* and *Y* are two ROIs, and X is activated before Y in the modeling results, is it also the case that *X* is activated before *Y* in the VSD data? If so, we count that ROI pair as a temporal agreement. If *X* and *Y* are activated in the same VSD frame, we count that pair as a case of insufficient temporal resolution. Finally, if *X* is activated after *Y* in the VSD data, we count that ROI pair as temporal disagreement.

## RESULTS

### Sensory-Specific Activation Cascades in the Mouse Cortex

**Figure 3.**
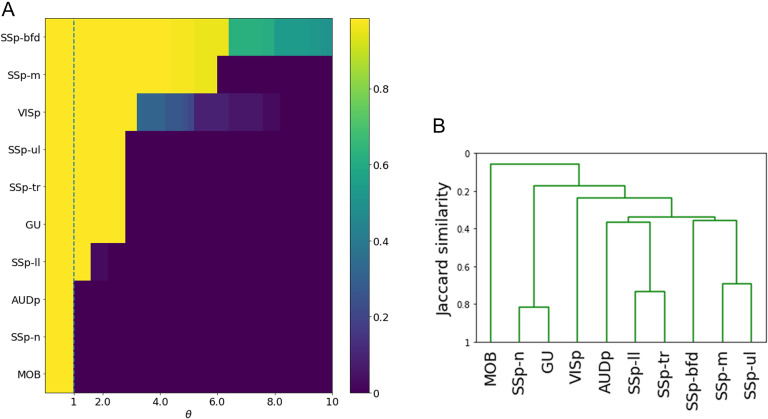
Effect of parameter *θ* on cascade size, and similarity between the 10 cascades. (A) Each row of the heat map shows the fraction of activated nodes after the stimulation of a single source, for different values of the threshold *θ*. The selected threshold is marked with the dashed vertical line. (B) Similarity between the 10 sensory cascades using the average-linkage hierarchical clustering method.

The ALT model requires the selection of a single parameter, the activation threshold *θ*. This threshold controls the *size of the cascade*, that is, the fraction of network nodes that become active after the activation of a source node. One may expect that as *θ* decreases towards zero, the size of the cascade becomes *gradually* larger. This is not the case, however. Figure 3A shows that as *θ* decreases we observe a rapid transition from the absence of a cascade (where only the source node is active) to a complete cascade, in which all nodes become active. This is true for all source nodes listed in Table SI-2.

The reason behind this rapid transition is the highly clustered topology of the mouse connectome. This property is often quantified by the clustering coefficient (Newman, 2018), which has an average value of 0.60 in the mouse connectome. This means that if a node *v* connects to two nodes *u* and *w*, there is a probability of 60% that there is an edge from *u* to *w* or in the opposite direction, “closing the triangle” formed by the three nodes *u*,*v*,*w*. So, if *v* is the source of the activation and *θ* is low enough so that *v* activates at least one of its neighbors, say *u*, it is highly likely that other neighbors of *v* receive input from *u* as well, increasing the chances that they will also get activated. The same argument applies to all other pairs of activated nodes—not only the source and its direct neighbors.

We choose *θ* so that the ALT model produces a complete cascade, for every source we consider (Beggs & Timme, 2012). This choice is motivated by experimental results (Mohajerani et al., 2013): At least some activity is detected in all cortical regions after sensory stimulation (visual, auditory, touch) in anesthetized mice. It may appear counterintuitive that visual stimulation, for example, can impact activity in regions associated with different sensory modalities (e.g., gustatory), but such interactions are possible through the many feedback connections in the connectome, and they are consistent with several prior studies that argue that there are no strictly unisensory regions, and that to some extent the entire cortex is a multisensory organ (Cappe & Barone, 2005; Falchier, Clavagnier, Barone, & Kennedy, 2002; Ghazanfar & Schroeder, 2006; Morrill & Hasenstaub, 2018).

Lower values of *θ* would also result in complete cascades. However, as *θ* decreases, the dynamics of the underlying cortical networks would move away from the critical boundary between sensitivity to internal or external stimulation and stability (Chialvo, 2010; Plenz & Thiagarajan, 2007).

Based on the previous considerations, we select *θ* = 0.98 asthe lowest value that results in a complete cascade across all sensory modalities. We have repeated the analysis for two more values of *θ* (0.90 and 0.95) without any significant changes in the results (see SI Section “Sensitivity to activation threshold *θ*”).

#### Similarity of Sensory Cascades.

How similar are the 10 sensory cascades predicted by the ALT model? The similarity between two cascades can be quantified using the Jaccard similarity metric. It is defined as the ratio of the common connections in two cascades over the total number of connections in those cascades.

After calculating the Jaccard similarity between every pair of cascades, we use an agglomerative hierarchical clustering algorithm to construct a dendrogram of the 10 cortical sensory cascades. This dendrogram was computed for three linkage methods: *average linkage* (the similarity between two clusters is based on the average similarity across all pairs of cascades in the two clusters), *single linkage* (based on maximum similarity), and *complete linkage* (based on minimum similarity). The resulting dendrograms are quite similar across the three linkage methods. Figure 3B shows the dendrogram with average linking—the two others are shown in Figure SI-3.

A first observation is that the olfactory cascade (originating at the main olfactory bulb [MOB]) is very different than all other sensory cascades—its similarity is less than 10% with the cluster of all other cascades. This is expected given that olfaction is quite different from all other sensory processes, it bypasses the thalamus, and MOB is the only primary sensory ROI in the mouse connectome that is not located in the isocortex (Johnson, Illig, Behan, & Haberly, 2000; Srinivasan & Stevens, 2017).

Interestingly, the two most similar cascades are the gustatory (GU) and the somatosensory cascade of the nose (SSp-n). Further, these two cascades are quite different from all others, including the rest of the somatosensory cascades. The somatosensory cascades are quite similar to each other and they tend to cluster as follows: trunk and lower limb (similarity of about 75%), mouth and upper limb (about 70%), while the whiskers produce a significantly different cascade than the previous four (similarity of about 40%). This organization mirrors the anatomical layout of the somatosensory regions. The auditory and visual cascades are also quite distinct from all other cascades—but not as dramatically different as olfaction.

#### Visual Cascade.

**Figure 4.**
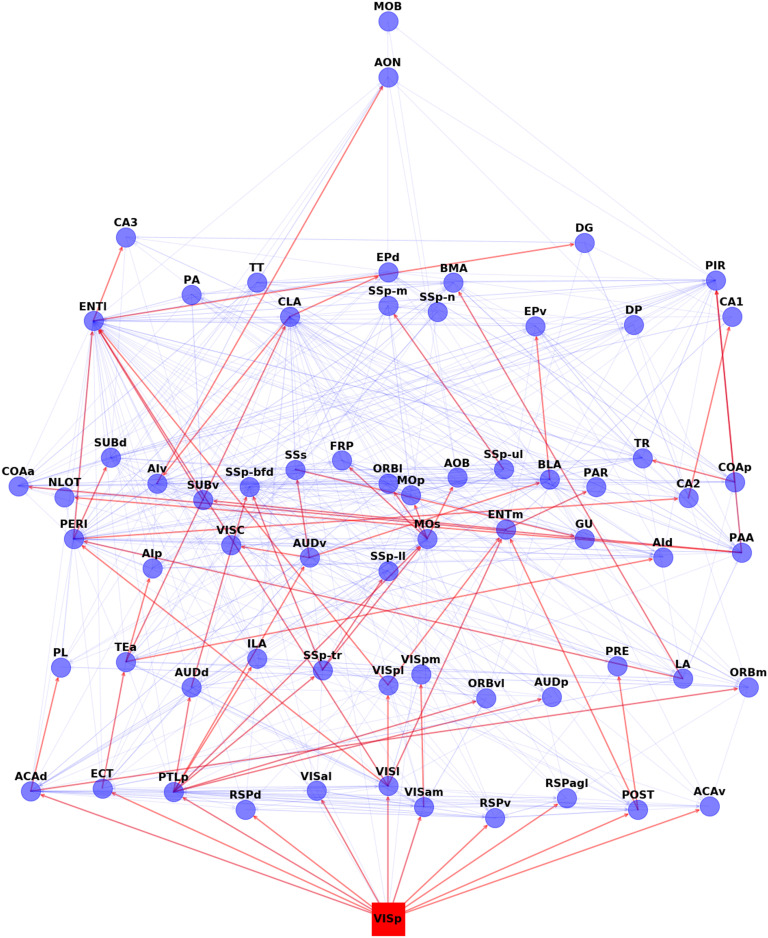
The visual activation cascade, according to ALT (*θ* = 0.98). The source for this cascade is the primary visual cortex (VISp). The red edges form the activation cascade, while the underlying blue edges show anatomical connections that do not participate in this cascade, those connections may be present in other sensory cascades or they may play a role in feedback (or second-order) interactions that are not captured by the “first ripple” scope of the ALT model. To help with the visualization, we place the nodes in eight layers, so that cascade edges only point from a layer to a higher layer (never to the same or lower layer). The vertical position of each node is slightly “jittered” to avoid cluttering due to anatomical connections between nodes of the same layer.

Figure 4 shows the complete activation cascade when the source of the stimulation is the primary visual cortex (VISp)—the corresponding cascades for the other sensory modalities are included in the Supporting Information (see SI Section “The ALT cascade of eachsensory source”). Note that the activation of the source triggers the activation of 11 other ROIs. Only a few of them, however, play a major role in extending the cascade to the rest of the network: ECT (ectorhinal), PTLp (posterior parietal association), VISl (lateral visual), and POST (postsubiculum). PTLp in particular causes the activation of seven more ROIs at the next step of the cascade. The activation of the claustrum (CLA), in this cascade, takes place through the sequence of ECT, followed by TEa (temporal association).

We emphasize that the hierarchical layout shown in Figure 4 is specific to each sensory modality and it represents the activation cascade from the corresponding source to the rest of the cortex. This notion of hierarchy should not be confused with the hierarchical organization of the cortex (Markov et al., 2013) that results from anatomical distinctions of intracortical connections (feedforward versus feedback, based on laminar markers; Harris et al., 2018). The latter is an anatomical hierarchical structure, it is not specific to any particular sensory modality, and it does not convey any functional information about how one ROI may be affecting another in the presence of a specific external or internal stimulation. An activation cascade, on the other hand, reveals the putative sequence and causal dependencies through which ROIs get activated after an initial activation at the source ROI.

The reader can find the activation cascade of each sensory source in SI Section “The ALTcascade of each sensory source.” It turns out that only about half the connections of the anatomical connectome appear in sensory activation cascades, and a quarter of the former appear in only one sensory cascade. In Section Figure SI-4 we examine: which anatomical connections are more important in terms of MSI? The results of that analysis suggest that sensory cascades spread through short connections connecting physically adjacent regions, rather than through the (relatively few) long connections that connect remote regions.

### Model Validation

We examine the validity of the ALT model predictions using functional imaging data during sensory stimulation experiments, as discussed in “Comparison of Modeling Results With Functional Dat” section. The question we focus on is the following: After stimulating a specific sensory modality (e.g., visual), if the ALT model predicts that an ROI *X* should be activated before an ROI *Y*, is it true that *X* gets activated before *Y* in the functional imaging data? When this is the case, we count the pair (*X*,*Y* ) as a temporal agreement. If *X* gets activated before *Y* in the ALT model but the opposite is true for the experimental data, we count (*X*,*Y* ) as a temporal disagreement. Because of the finite temporal resolution in the experimental results (each frame is sampled every 7 ms roughly), there are also cases where *X* and *Y* appear to be activated during the same frame, while the model always predicts a temporal difference between two activations. When that is the case, we count (*X*,*Y* ) as a case of insufficient temporal resolution.

**Figure 5.**
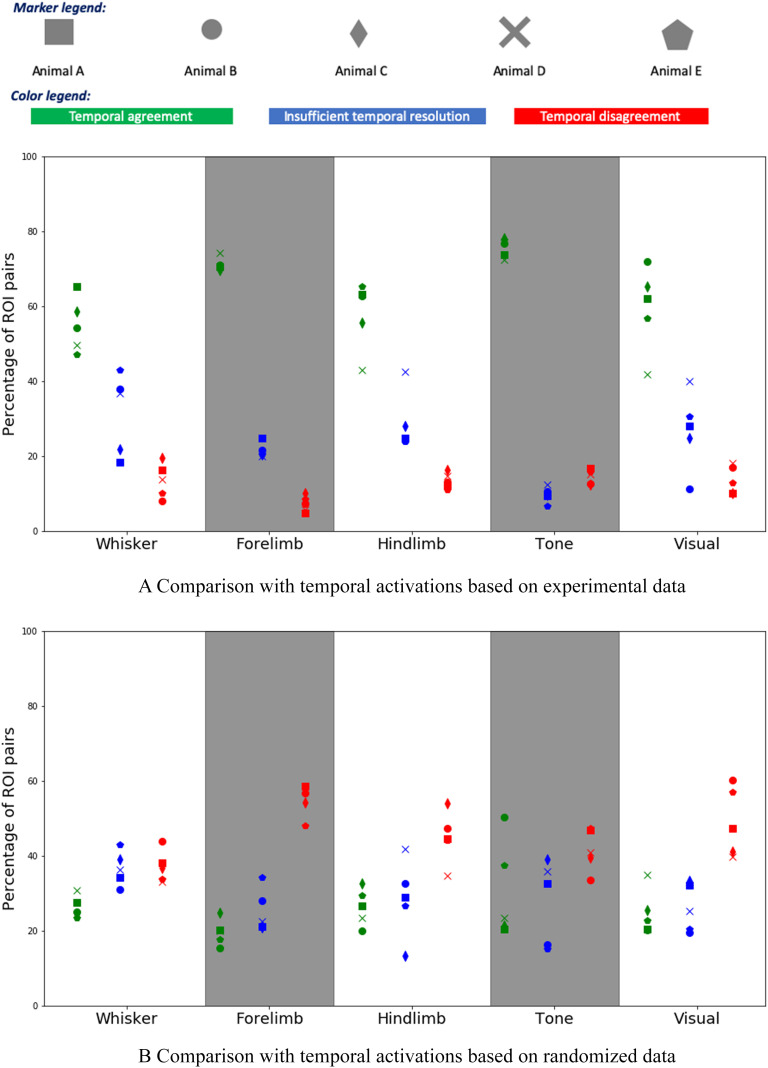
Comparison between model-based and experimental temporal ordering of ROI activations. (A) The y-axis shows the percentage of (*X*,*Y* ) ROI pairs that show temporal agreement (green), temporal disagreement (red), and insufficient temporal resolution (blue) between the activation order of *X* and *Y* in the modeling results and the mouse experiments. The plot shows results for five animals and for five sensory stimulations (a touch at the whiskers, forelimb, and hindlimb, as well as an auditory and a visual stimulation). (B) The same comparison, but here we have randomized the ROIs that are active during each frame, preserving the number of ROI activations in each frame.

Figure 5A shows the percentage of (*X*,*Y* ) ROI pairs that show temporal agreement, temporal disagreement, and insufficient temporal resolution between the activation order of *X* and *Y* in the modeling results and the mouse experiments. The plot shows results for five animals and for five sensory stimulations. Even though the variability across animals is considerable, we observe that the percentage of temporal agreement pairs, averaged across the five animals, is higher than 50% and it varies between 55% to 80% depending on the sensory modality. On the other hand, the corresponding percentage of temporal disagreement is less than 10%–20%, depending on the stimulation. In the rest of the cases, the temporal resolution is not sufficient.

Note that the VSD experiments were repeated 10 times (for each animal, and for each sensory stimulation), as described in (Mohajerani et al., 2013). In our analysis however, we only have access to the aggregated results of those 10 experiments, and so we have a single sample for each animal and for each sensory stimulation. This is a limitation of the current analysis. To quantify the variability of these results, we show five point estimates, one for each animal.

We also compare the ALT modeling results with a randomized sequence of experimental activations in which we preserve the number of ROIs that are activated in each frame but assign random ROI activations during each frame. This comparison shows that the ALT model has significant prediction power on the temporal sequence of ROI activations, relative to a randomized baseline.

Finally, we analyzed the temporal disagreement cases between VSD experiments and modeling results to examine whether certain brain regions, or pairs of regions, are overrepresented in those disagreements (see SI Section “Analysis of disagreement cases between VSD data andALT modeling results”). The main result of that analysis is that the ROIs with highest disagreement cases appear at the boundary of the cortical surface at the VSD datasets and they are only partially visible. So, it is likely that the VSD data may not accurately capture the time at which those boundary regions are activated after each stimulation.

### Core ROIs and Hourglass Architecture

**Figure 6.**
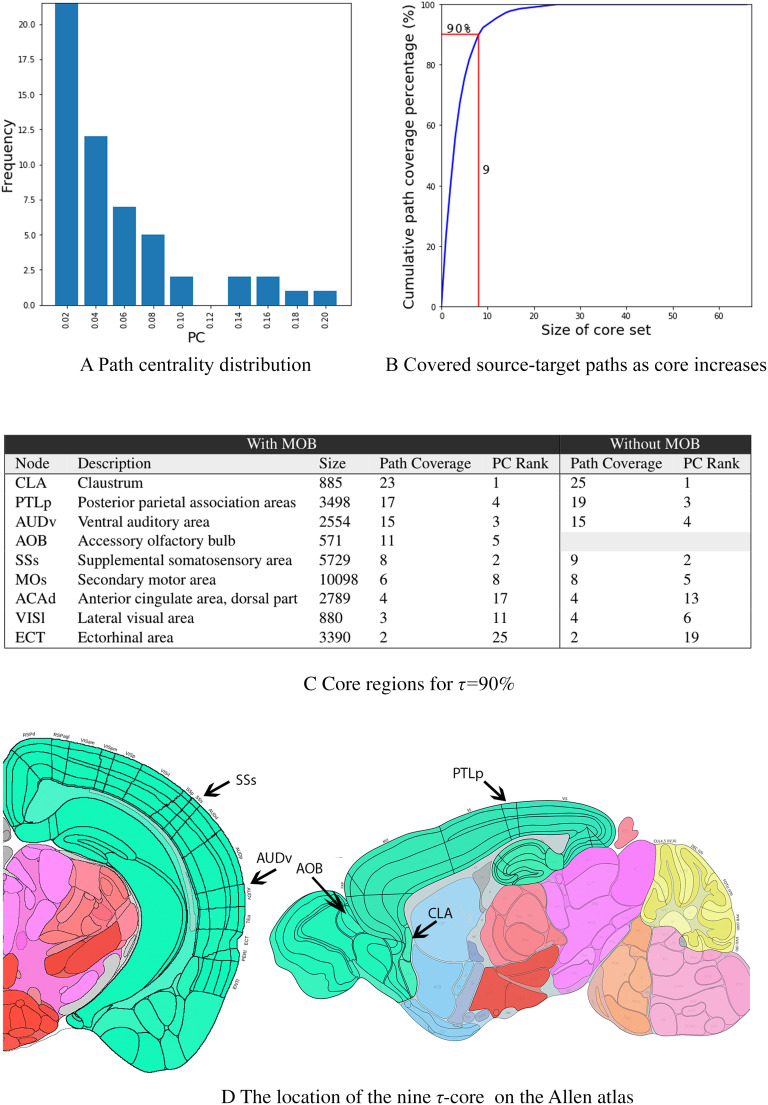
Path centrality and *τ*-core analysis. (A) Path Centrality (PC) histogram for the 67 regions in *N*_*c*_, considering all source-target paths across the 10 activation cascades. (B) Cumulative path coverage by the top *X* core nodes for *X* = 1, ⋯ ,67. Nine regions are sufficient to cover *τ* = 90*%* of all paths. (C) Core regions for *τ* = 90% also showing the path coverage contributed by each of them and its path centrality rank. (D) The location of the top five core regions.

In this section, we analyze the collection of 10 activation cascades (one cascade for each source) using the network analysis approach described in Section “Analysis of ActivationCascades.” The total number of source-target paths in the 10 cascades is 560. The path centrality distribution, which captures how many activation paths traverse each node, is shown in Figure 6A. Almost half of the nodes have very low path centrality (2% or less). On the other hand, there are four nodes with much larger path centrality—each of them covering about 20% of the source-target paths in the collection of activation cascades. These ROIs are the CLA (claustrum), SSs (supplemental somatosensory), PTLp (posterior parietal association), and AUDv (ventral auditory) areas.

Some activation paths can traverse more than one of these highly central regions. For this reason, we compute the minimal set of nodes that cover a given fraction *τ* of all source-target paths, that is, what is referred to as **τ*-core* (Sabrin & Dovrolis, 2017). Figure 6B shows the fraction of covered source-target paths as we increase the size of the *τ*-core. The “knee-shaped” shape of this curve suggests that a small set of nodes is sufficient to cover almost all source-target paths in the activation cascades, forming a bottleneck in the MSI process. For instance, a set of nine nodes is sufficient to cover more than 90% of all source-target paths. These nine ROIs account for 14.7% of the brain volume of the ROIs we consider in the network *N*_*c*_.

The small size of the *τ*-core, relative to the size of the network, suggests that the cortex follows an *hourglass architecture*, in which the sensory information from different modalities is first *integrated* (other terms could be “encoded” or “compressed”) by a small set of *τ*-core ROIs that form the “waist” (or bottleneck) of the hourglass. Those *τ*-core ROIs then drive a large number of downstream ROIs that presumably operate on multisensory information and contribute in higher level cognitive processes. The benefit of an hourglass architecture is that it reduces the dimensionality of the input, computing a more compact intermediate-level sensory representation at the waist of the hourglass.

The *τ*-core nodes for *τ* = 90% are listed in Figure 6. Together with the percentage of *additional* source-target paths that each node contributes to the *τ*-core (path coverage), the figure also shows the path centrality (PC) rank of that node. As expected, the node with the highest PC is the first node in the *τ*-core. After that point, the order in which nodes join the *τ*-core does not follow their PC ranking. The top three nodes (CLA, PTLp, AUDv) are sufficient to collectively cover about 60% of the activation paths. Note that none of these *τ*-core ROIs are a primary sensory region (i.e., a source node). Instead, they are either ROIs that are not typically associated with a single sensory modality (CLA, PTLp, ACAd, ECT) or they are ROIs that are often thought of as secondary or supplemental to a certain sensory modality (AOB, AUDv, SSs, MOs, or VISl). If we exclude the MOB cascade, the only difference is that the *τ*-core will not include the AOB region.

The previous analysis is based on the path centrality metric and the *τ*-core notion. In SI Section “Comparison with other centrality metrics and other network core notions,” we examine the correlation between the path centrality metric and other, more commonly used centrality metrics. That section also compares the nodes in the *τ*-core with other core node sets (rich-club and Rombach core-periphery).

Further, in SI Section “Activation cascades when two sensory sources are activated simultaneously,” we extend the previous analysis in the case that two source nodes become simultaneously active. It turns out that the core nodes are the same with the single-source case, except that the anterior cingulate area—dorsal part (ACAd) and the ectorhinal area (ECT)—are replaced by the perirhinal area (PERI).

### Location of *τ*-Core Nodes in Activation Cascades

#### Location Relative to Sources.

In this section, we first investigate the *topological location* (rather than anatomical location) of the *τ*-core nodes relative to the source of each activation cascade. Are the *τ*-core nodes, which form the waist of the hourglass architecture, closer to the sources or targets of the activation cascades? And how does their location compare with the location of the sources and of other cortical ROIs? These questions are related to recent experimental work suggesting that cross-modal representations are constructed at early stages of the sensory information flow (Cayco-Gajic & Sweeney, 2018).

**Figure 7.**
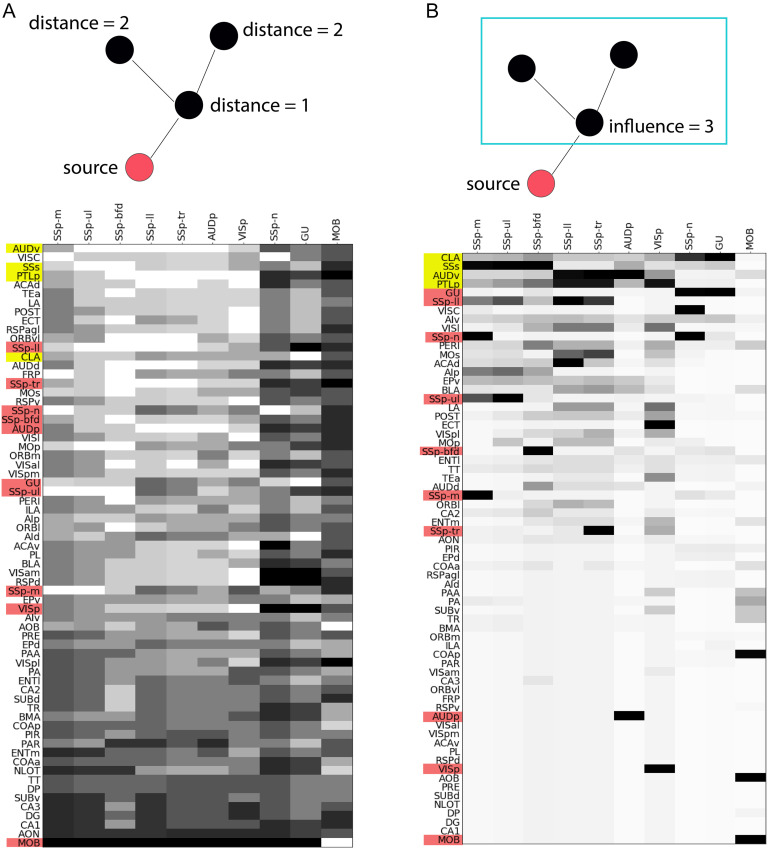
Location-related metrics. In both matrices, a column represents one of the 10 activation cascades, originating at the node shown at the top of the column. (A) Each row represents the *source-distance* of the corresponding node from the source of that column’s cascade. White denotes a distance of one hop, while black denotes the maximum distance for that cascade. Rows are ordered in terms of the average distance (in number of hops) of the corresponding node from the sources of all activation cascades (excluding the MOB cascade, which is very different). (B) Each row represents the *influence* of the corresponding node, that is, the number of nodes that are reachable from that node in the activation cascade that the column represents. White denotes an influence of one (only that node), while black denotes an influence that covers all network nodes. Rows are ordered in terms of the average influence of the corresponding node across all activation cascades (excluding the MOB cascade).

We focus on the top four *τ*-core nodes (CLA, PTLp, AUDv, SSs), which collectively cover about *τ* = 70% of all source-target paths (see Figure 6). Figure 7A visualizes in gray scale the location of each node (matrix row) in each activation cascade (matrix column). Each source node (represented with red) is obviously at a distance of 0 in its own activation cascade. Note however that source nodes can be at a much larger distance from sources of other activation cascades. For instance, the primary visual cortex (VISp) appears at the maximum distance in the nose (SSp-n) and gustatory (GU) cascades.

The four *τ*-core nodes we consider (represented with yellow rows) are relatively close to all source nodes: AUDv has the lowest average distance to all of the primary source areas, while SSs and PTLp are ranked as third and fourth. The claustrum (CLA) is slightly farther away from the sources, ranked 13th (out of 67) in the previous ranking. If we consider a higher value of *τ* = 90%, the additional *τ*-core nodes (MOs, ACAd, VISl, and ECT) are ranked as 17th, 5th, 22nd, and 9th in terms of their average distance from sources. In summary, all *τ*-core nodes appear in the top one third of the distance ranking, and so they are closer to the sources of the hourglass architecture than to its targets.

#### Location Relative to Targets—Influence.

Another way to examine the location of a node *v* in the hourglass architecture is in terms of how many nodes appear in activation paths downstream of *v*—a metric that we refer to as the *influence* of *v*. Figure 7B visualizes in gray scale the influence of each node (matrix row) in each activation cascade (matrix column). The source of a specific cascade has, by definition, maximal influence (i.e., all network nodes) in its own cascade—but it may have a much lower influence in other cascades. Indeed, the influence of source ROIs (shown in red) does not seem to follow a coherent pattern: The gustatory (GU) and somatosensory area of the lower limb (SSp-ll) are sources with high influence but the primary visual cortex (VISp), the primary auditory cortex (AUDp), and the main olfactory bulb (MOB) are sources with low influence in other cascades.

On the other hand, the four most important *τ*-core nodes (CLA, PTLp, AUDv, and SSs) also occupy the top four positions in terms of influence. The next four *τ*-core nodes (MOs, ACAd, VISl, and ECT) have high influence as well, ranked as 12th, 13th, 9th, and 20th, respectively.

Combining the previous observations about the influence of *τ*-core nodes as well as their distance from sources, we can summarize our findings as follows: *τ*-core nodes are close to most sensory sources and they also influence the activation of many downstream nodes. These two features place *τ*-core nodes at a location that allows them to both integrate sensory information from different sources as well as to use that integrated information in driving many downstream ROIs.

### Robustness of *τ*-Core Nodes

In this section, we examine the robustness of the previous results regarding the *τ*-core when we randomize the edges and weights of the underlying connectome. We also examine whether the length and/or weights of these connections are responsible for the hourglass effect and for the specific regions that form the *τ*-core.

We create ensembles of random connectomes, derived from the mouse connectome in four different ways:

1.  Randomize the weight assigned to each edge, reallocating the weights of the original connectome across randomly selected connections but maintaining the topology.
2.  Randomize the physical length of each edge (and thus its communication delay in the ALT model), again reallocating randomly the lengths of the original connections but maintaining the topology.
3.  Randomize both the weights and the lengths assigned to each edge, as previously mentioned. We do not maintain any correlation between weights and lengths.
4.  Randomize the connectome’s topology by swapping connections between randomly selected pairs of nodes. This randomization method preserves the in-degree and out-degree of each node.

**Figure 8.**
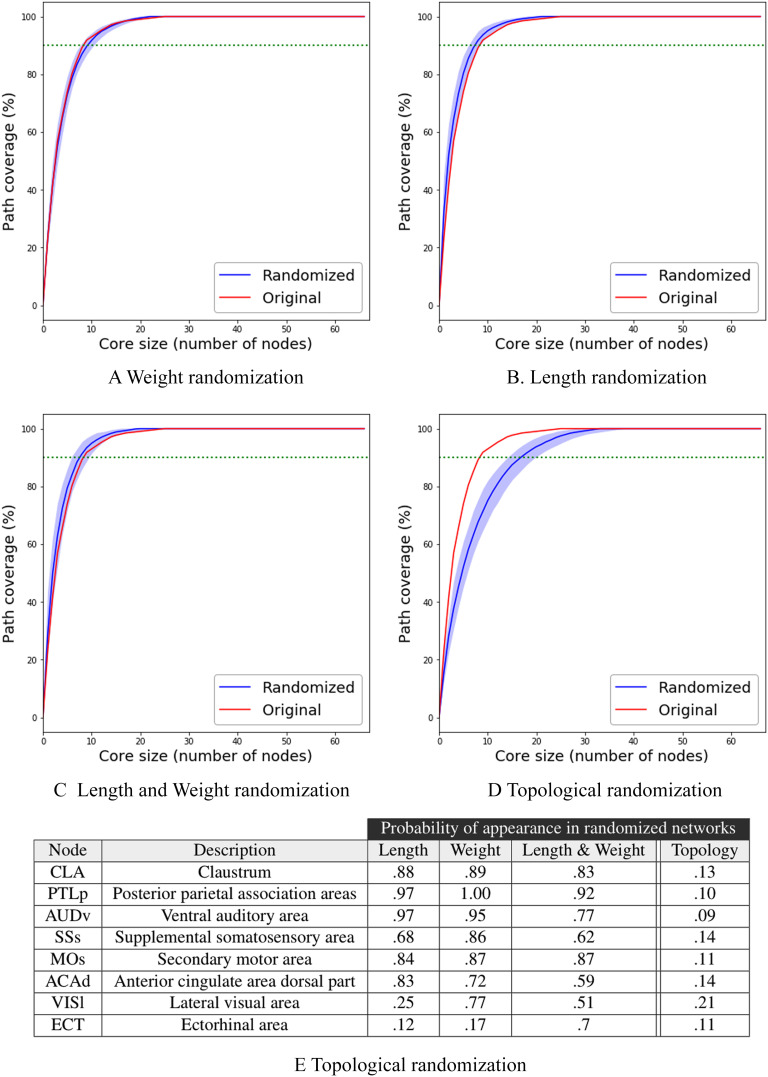
Robustness results. The effect of different connectome randomization methods on the core size. Light blue shade marks the 5% to 95% values among 100 randomization runs, while the solid blue line is the median of these runs. The red line represents the *τ*-core size for the original connectome. The dotted green line marks the *τ*-core size for *τ* = 90%. The table at the bottom shows the fraction of random networks that include each of the eight *τ*-core nodes.

Figures 8A–8D focus on the first three randomization methods: weights, lengths, and their combination. In all cases, the *τ*-core size of the original network is contained in the 5% confidence interval of 100 randomized networks. In other words, the weights and physical lengths of the connectome’s connections do not play a significant role in the number of *τ*-core nodes, for any value of *τ*

On the other hand, when we randomize the topology of the connectome, the *τ*-core size doubles in size when *τ* = 90%: from 9 nodes in the original network to 18. Additionally, it takes about half of the entire network to cover all activation paths in the collection of 10 A-DAGs. So, it is the graph structure of the connectome (i.e., its topology) that leads to a small *τ*-core size, not the weight and/or length of the connections.

Even though the weight and length of the connections do not have a strong effect on the *τ*-core size, do they affect the identity of the ROIs that participate in that *τ*-core? To answer this question, Figure 8E shows the fraction of random networks that include each of the nine *τ*-core nodes in Figure 6C. The claustrum (CLA), for instance, appears in the MSI *τ*-core of 88% of the networks that have randomized connection lengths (89% for randomized weights) but in only 13% of the networks that have randomized topology. The results are similar for the top six MSI *τ*-core nodes: They appear in the MSI *τ*-core of most randomized networks when we randomize connections weights and/or lengths, but they rarely appear in the MSI *τ*-core when we randomize the topology. For the last two MSI *τ*-core nodes (VISl and ECT) their membership in the MSI *τ*-core is *not* as robust: Randomizing connection lengths has a major effect in the appearance of VISl in the *τ*-core, and randomizing any aspect of the network has a major effect in the appearance of ECT in the *τ*-core.

Finally, we have applied the ALT model on the four types of randomized connectomes (randomized weights, lengths, weights and lengths, and topology) and compared the resulting cascades with the corresponding VSD-based visual cascade. The results of this comparison (see SI Section “Modeling versus experimental results on randomized connectomes”) confirm that the randomization of lengths and/or weights has a minimal effect, while topological randomization causes a major reduction in the percentage of ROI pairs that show “temporal agreement” between modeling and experimental results.

### Effect of Subcortical Regions on Cortical Cascades

In this section, we examine the effect of subcortical regions on cortical sensory cascades. The cascade sources remain the same 10 cortical primary sensory regions. The network on which we apply the ALT model, however, now covers the entire mouse connectome. So, the activation paths that originate from a source can now also traverse both cortical and subcortical regions

One first observation is that using the same activation threshold value (*θ* = 0.98) as in the cortical network, the cascade from every source covers the entire brain, including all subcortical regions. In other words, the activation threshold is sufficiently low to include all subcortical regions in the sensory cascades.

When we consider the whole connectome, the activation paths that originate from a certain source node can be classified in three major groups: *C-paths* (traversing only cortical regions), *CS-paths* (crossing the cortical-subcortical boundary once and terminating at subcortical regions), and *CSC^+^-paths* (crossing the cortical-subcortical boundary twice or more but terminating at cortical regions.^3^

Across all 10 sources, *C-paths* account for 25%, *CS-paths* for 65%, and *CSC^+^-paths* for about 10% of all paths. In the rest of this section, we focus on the union of *C-paths* and *CSC^+^-paths* because, first, the validation of the ALT-based cascades with VSD data is only applicable to cortical regions, and second, we are primarily interested in multisensory integration in the cortex.

To examine whether the inclusion of subcortical regions affects the sensory cascades in the cortex, we examine, for a given source (e.g., visual stimulation), how similar the ALT-based cascades that terminate at cortical nodes are, with and without the *CSC^+^-paths*. Note that we do include *CS-paths* because they do not participate in that portion of the cascades. At the visual cascade, for instance, the Kendall *τ* coefficient between the two activation sequences is 92% (*p* value < 10^−27^). The correlations are similarly high for the other sources. This suggests that the inclusion of subcortical regions does not significantly affect the timing sequence at which cortical regions get activated after a sensory stimulus.

We also compute the *τ*-core for the union of *C-paths* and *CSC^+^-paths*. The results are shown in Table 1 for *τ* = 90%. The core is now slightly larger (14 nodes instead of the 9 nodes in Figure 6C). The top six nodes are the same as in Figure 6C but in different order. The core now also includes three subcortical regions: The reticular nucleus (RT) and the peripeduncular nucleus (PP) in the thalamus, and the magnocellular nucleus in the pallidum.

**Table 1.** Core nodes for *τ* = 90% when we include the subcortical regions in the cortical sensory cascades.

Node	Description	Region	Size	Path Coverage	PC Rank
SSs	Supplemental somatosensory area	Isocortex	5,729	18	1
PTLp	Posterior parietal association areas	Isocortex	3,498	16	4
AOB	Accessory olfactory bulb	Olfactory areas	571	15	3
CLA	Claustrum	Cortical subplate	885	10	2
AUDv	Ventral auditory area	Isocortex	2,554	9	5
MOs	Secondary motor area	Isocortex	10,098	4	10
RT	Reticular nucleus of the thalamus	Thalamus	1,395	4	25
ACAd	Anterior cingulate area, dorsal part	Isocortex	2,789	3	18
VISl	Lateral visual area	Isocortex	880	3	12
PP	Peripeduncular nucleus	Thalamus	111	2	53
PERI	Perirhinal area	Isocortex	1,412	2	8
MOp	Primary motor area	Isocortex	11,760	2	19
MA	Magnocellular nucleus	Pallidum	376	2	59
ENTm	Entorhinal area, medial part,	Hippocampal	3,106	1	13
	dorsal zone	formation			

The appearance of RT in this list is not surprising: This region is known to be highly interconnected with many cortical areas, and it provides a strong modulatory input to cortex that drives the brain into different attentional states (Halassa et al., 2014) and gates the flow of many different sensory streams to cortex (Zikopoulos & Barbas, 2007).

## Discussion

This work built a suite of modeling and analysis tools to study multisensory integration from the perspective of communication dynamics. We chose to analyze the mouse connectome because of the availability of a detailed mesoscale anatomical map and because the problem of multisensory integration is relatively well studied in rodents (Meijer, Mertens, Pennartz, Olcese, & Lansink, 2019). In particular, we analyzed the cortical brain network from the Allen Mouse Brain Connectivity Atlas (Oh et al., 2014) and focused on “early” dynamics of sensory integration. By *early* we specifically mean the first wave of cortical activity, starting from primary sensory areas and propagating to the whole hemisphere. This point differentiates our work from earlier models of multisensory integration (Ripp et al., 2018; Zamora-López, Zhou, & Kurths, 2010).

The underlying anatomical pathways recruited by diverse sensory modalities (e.g., visual, auditory, somatosensory) branch out rapidly and become increasingly complex as they reach the higher associative cortical areas (Mohajerani et al., 2013). To capture the nonlinear and asynchronous nature of these dynamics, we used a diffusion model that can capture both nonlinearity and communication delays, called asynchronus linear thresholding (ALT). While this model is surprisingly simple, we found that ALT can closely recapitulate sensory cascades when compared with VSD datasets from sensory stimulation experiments on mice (Mohajerani et al., 2013).

ALT is a phenomenological model that aims to describe (but not explain mechanistically) the diffusion of information at the ROI level. As such, it does not model the underlying mechanisms of neuronal communication through chemical or electrical synapses, and it cannot capture more complex dynamics such as sustained oscillations at certain frequencies or feedback from a newly activated region back to regions that were activated earlier. Modeling such dynamics would require more elaborate neural mass models (Destexhe & Sejnowski, 2009). The validation of such models, however, would require whole-brain neuroimaging data of fine spatial and temporal resolution (higher temporal resolution than fMRI and higher spatial resolution that EEG). Additionally, those more complex models may not be necessary when the goal is to map the feedforward initial propagation of brain activity after a sensory stimulation.

Similar to neuroimaging modalities of similar spatial resolution (such as task-based fMRI), ALT aims to capture how a certain activity, namely the stimulation of a primary sensory region, causes the activation of other brain regions. A difference with fMRI or MEG, however, is that the resulting activation cascades can be analyzed to infer interactions between ROIs that participate in the cascade. Additionally, ALT depends on the communication delays and weights of the connections between brain regions. Thus, it produces a timeline of activation events, one for each ROI that participates in the cascade. The length of the time interval between activation events, at least in relative terms, can be compared with experimental results from neuroimaging modalities such as VSD (used in this study) or calcium imaging.

We emphasize that ALT is not the only plausible modeling approach to study the diffusion of sensory information in the brain. There are several other models in the literature that attempt to capture communication dynamics in the brain based on shortest path lengths, search information (Goñi et al., 2014), navigation (Seguin, van den Heuvel, & Zalesky, 2018), diffusion efficiency (Goñi et al., 2013), and others (Avena-Koenigsberger, Misic, & Sporns, 2018). Which of these models produces the most accurate results, compared with experimentally observed activation cascades, is an open question that deserves further investigation.

A major result of this study is that relatively few cortical regions are responsible for integrating almost all sensory information. This finding supports the idea that multisensory integration is performed through an hourglass architecture. The benefit of an hourglass architecture is that first it reduces the input dimensionality of the sensory stimulus at few intermediate-level modules that reside at the hourglass waist. Second, it reuses those compressed intermediate-level representations across higher level tasks, reducing redundancy between the latter. The hourglass analysis framework was first developed in Sabrin and Dovrolis (2017), and it has been recently applied in the connectome of *C. elegans* (Sabrin, Wei, van den Heuvel, & Dovrolis, 2020). There are two fundamental differences in our study: (a) We rely on communication dynamics, while Sabrin et al. (2020) uses a predefined set of “routing mechanisms” to construct ensembles of sensory pathways. (b) The sources and targets of the *C. elegans* cascade are the sensory and motor neurons, respectively. Nonetheless, it is interesting that the hourglass architecture emerges in both studies. One possibility is that this architecture is selected by evolution because it drives a network towards reusing a small set of intermediate functions in constructing a range of redundant output functions.

Rather than studying each sensory cascade in isolation, our analysis framework is based on the combination of all sensory cascades. Comparative and competitive cascades have been studied in Mišić et al. (2015) to quantify the combined effect of multiple cascades, using simultaneously activated source nodes. Sensory stimuli of different modalities, however, do not need to arrive at the cortex simultaneously in order to be integrated (Noel, Łukowska, Wallace, & Serino, 2016). Different sensory stimuli travel at different speeds through body receptors (Zhou et al., 2019). The analysis framework that we followed constructs unisensory cascades and merges their activation paths. We have also experimented with cascades originating from two simultaneously activated sources (see SI Section “Activation cascades when two sensorysources are activated simultaneously”). The hourglass architecture is still observed in that case, and the core nodes are mostly the same.

The claustrum is known for its anatomical uniqueness (Baizer, Sherwood, Noonan, & Hof, 2014) and its precise function has been enigmatic (Mathur, 2014; Van Horn, 2019). The late F. Crick has hypothesized that the claustrum may be a potential gateway to consciousness (Crick & Koch, 2005). More recent results have demonstrated that the claustrum is important in gating sensory information, and in attention mechanisms in visual perception (Van Horn, 2019). These and other studies provide strong evidence that the claustrum is a crucial node for multisensory integration. Our findings are in agreement with this growing body of work.

The posterior parietal cortex is the second most important region for MSI, according to our analysis. This stems from its strong and immediate connectivity to primary sensory regions and its projections to motor areas. PTLp’s connectivity points to its role in integrating sensory information to direct immediate motor commands (Whitlock, 2017). The experiments of Nikbakht, Tafreshiha, Zoccolan, and Diamond (2018) provide direct evidence that PTLp is multisensory at both the behavioural and the neurophysiological levels, and it provides sensory-independent information about the orientation and categorization of objects in the environment. We refer the reader to Mohan, de Haan, Mansvelder, and de Kock (2018) for further discussion.

Three additional core nodes have been associated with specific sensory modalities in the past: ventral auditory area (AUDv), supplemental somatosensory area (SSs), and lateral visual area (VISl). However, all of them have also been implicated to some extent with multisensory processing. For instance, Hishida, Kudoh, and Shibuki (2014) found that activity propagating to the parietal association area passes through the ventral auditory region, irrespective of the sensory stream source (visual, auditory, or somatosensory). Similarly, Menzel and Barth (2005) suggest that SSs has a major role in bringing context to the sensory pathways (Romo, Hernández, Zainos, Lemus, & Brody, 2002). VISl is on the dorsal visual stream is associated mostly with spatial location action guidance (Marshel, Garrett, Nauhaus, & Callaway, 2011), while the other secondary visual areas participate in the ventral stream (Hebart & Hesselmann, 2012).

Various nuclei in the thalamus or the superior colliculus are known to be crucial in MSI (Stein, Stanford, & Rowland, 2014). An integrated model of both cortical and subcortical activity, in the context of sensory integration, may help in the future to explain the complex cortico-thalamic feedback mechanisms (Jiang, Wallace, Jiang, Vaughan, & Stein, 2001; Tyll, Budinger, & Noesselt, 2011). Disentangling such communication dynamics requires working at finer spatial resolutions, and including distinct cortical layers (laminar divisions) (Goulas, Zilles, & Hilgetag, 2018). Incorporating high-resolution (Knox et al., 2018) and cell-type-specific (Harris et al., 2018) information about connectivity in our pipeline is an interesting direction that can allow segregation of different communication channels. By increasing the spatial resolution and the complexity of the model dynamics, we may be able to capture more complex population-level activity patterns (e.g., Wilson-Cowan [Destexhe & Sejnowski, 2009]) and better understand the role of mesoscale network topology in constructing a coherent perceptual state from raw sensory streams.

## ACKNOWLEDGMENTS

The authors are deeply grateful to Professor Majid Mohajerani for providing the VSD data used in this study, and his student Zahra Rezaei for her help with that dataset.

## SUPPORTING INFORMATION

upporting information for this article is available at https://doi.org/10.1162/netn_a_00164.

## AUTHOR CONTRIBUTIONS

Kamal Shadi: Conceptualization; Data curation; Formal analysis; Investigation; Methodology; Software; Visualization. Eva Dyer: Conceptualization; Supervision; Visualization; Writing - Review & Editing. Constantine Dovrolis: Conceptualization; Data curation; Formal analysis; Funding acquisition; Investigation; Methodology; Project administration; Resources; Supervision; Validation; Visualization; Writing - Original Draft; Writing - Review & Editing.

## FUNDING INFORMATION

Constantine Dovrolis, Defense Sciences Office, DARPA (http://dx.doi.org/10.13039/100006502), Award ID: HR0011-18-2-0019. Eva Dyer, National Science Foundation (http://dx.doi.org/10.13039/100000001), Award ID: IIS-175587.

## Notes

^1^We use the terms node, region, and ROI (region of interest) interchangeably.^2^We have repeated the analysis for other *p* values in the range 0.01-0.1 – see SI Section “Connectome filtering”.^3^The fraction of paths that cross the cortical-subcortical paths twice or more and terminate at the subcortex is negligible.

## TECHNICAL TERMS

Asynchronous linear threshold (ALT) model: A network diffusion model in which a node becomes active if the cumulative weight of incoming connections from active neighbors is more than a given threshold. Each connection can also have a distinct propagation delay.
Network diffusion: A dynamic process through which the exogenous activity at one or more seed nodes spreads, at least partially, to the rest of the network through neighboring nodes.
Activation cascade: A directed acyclic graph that shows the network diffusion process from a given source node that corresponds to a primary sensory brain region.
Directed acyclic graph (DAG): A directed network without cycles. A DAG always includes one or more source nodes (no incoming edges) and one or more terminal nodes (no outgoing edges).
Path centrality: Given a set of activation cascades (one for each sensory source), the path centrality of a node is the total number of source-to-target paths, across all activation cascades, that traverse that node.
τ-core: Given a set of activation cascades, the *τ*-core is a set of nodes (brain regions) that collectively cover at least a fraction *τ* of all source-to-target paths.
Hourglass architecture: Given a set of activation cascades, the corresponding network has an hourglass architecture if almost all source-to-target paths go through a small number of intermediate nodes (the waist of the hourglass).
Mesoscale network: A graph that models the structural connectivity between brain regions, typically at the spatial resolution of few hundreds of micrometers.
