# Supplementary material for: Multisensory integration in the mouse cortical connectome using a network diffusion model
Source: Netw Neurosci. 2020 Nov 1;4(4):1030–54. doi: 10.1162/netn_a_00164 (PMC7655044; doi:10.1162/netn_a_00164)
Supplement: Supplementary file 1 [file netn-04-1030-s001.pdf]

Shadi, K., Dyer, E. & Dovrolis, C. (2020). Supporting information for "Multi-sensory integration in the mouse cortical connectome using a network diffusion model." *Network Neuroscience*, *4*(4), 1030-1054.
https://doi.org/10.1162/netn_a_00164

SUPPLEMENTARY INFORMATION (SI)

Connectome filtering

We have repeated the analysis for the following p-values: $0.01, 0.02, \cdots 0.1$. When the p-value is smaller than 0.03, the network becomes disconnected. When the p-value is higher than 0.04, the core nodes remain the same as in Table 6c.

Figure SI-1 shows the network density for different p-values (left) as well as the connection density (edge weight) versus p-value for each structural connection among the 67 ROIs we consider (right).

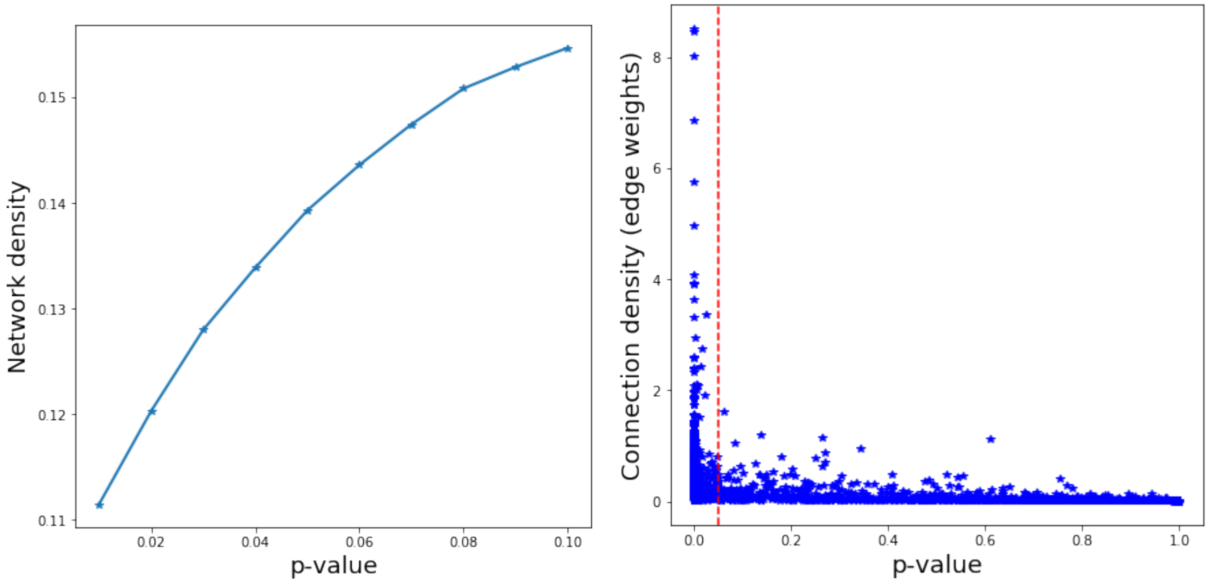

Figure SI-1: *Connectome filtering.* **Left:** Network density versus edge p-value. **Right:** Connection density (edge weight) versus edge p-value

All ROIs in cortical mouse connectome

In most of the paper (with the exception of Section) we consider the 67 ROIs that cover the four components of the cerebral cortex: *isocortex, olfactory areas, hippocampal formation, and cortical subplate*. They are listed in Table SI-1.

Table SI-1: *The list of 67 ROIs in the cortical portion of the mouse connectome.*

Node acronym	Node name	Node major region
ACAd	Anterior cingulate area, dorsal part	Isocortex
ACAv	Anterior cingulate area, ventral part	Isocortex
AId	Agranular insular area, dorsal part	Isocortex
AIp	Agranular insular area, posterior part	Isocortex
AIv	Agranular insular area, ventral part	Isocortex
AOB	Accessory olfactory bulb	Olfactory Areas
AON	Anterior olfactory nucleus	Olfactory Areas
AUDd	Dorsal auditory area	Isocortex
AUDp	Primary auditory area	Isocortex
AUDv	Ventral auditory area	Isocortex
BLA	Basolateral amygdalar nucleus	Cortical Subplate
BMA	Basomedial amygdalar nucleus	Cortical Subplate
CA1	Field CA1	Hippocampal Formation
CA2	Field CA2	Hippocampal Formation
CA3	Field CA3	Hippocampal Formation
CLA	Claustrum	Cortical Subplate
COAa	Cortical amygdalar area, anterior part	Olfactory Areas
COAp	Cortical amygdalar area, posterior part	Olfactory Areas
DG	Dentate gyrus	Hippocampal Formation

DP	Dorsal peduncular area	Olfactory Areas
ECT	Ectorhinal area	Isocortex
ENTl	Entorhinal area, lateral part	Hippocampal Formation
ENTm	Entorhinal area, medial part, dorsal zone	Hippocampal Formation
EPd	Endopiriform nucleus, dorsal part	Cortical Subplate
EPv	Endopiriform nucleus, ventral part	Cortical Subplate
FRP	Frontal pole, cerebral cortex	Isocortex
GU	Gustatory areas	Isocortex
ILA	Infralimbic area	Isocortex
LA	Lateral amygdalar nucleus	Cortical Subplate
MOB	Main olfactory bulb	Olfactory Areas
MOp	Primary motor area	Isocortex
MOs	Secondary motor area	Isocortex
NLOT	Nucleus of the lateral olfactory tract	Olfactory Areas
ORBl	Orbital area, lateral part	Isocortex
ORBm	Orbital area, medial part	Isocortex
ORBvl	Orbital area, ventrolateral part	Isocortex
PA	Posterior amygdalar nucleus	Cortical Subplate
PAA	Piriform-amygdalar area	Olfactory Areas
PAR	Parasubiculum	Hippocampal Formation
PERI	Perirhinal area	Isocortex
PIR	Piriform area	Olfactory Areas
PL	Prelimbic area	Isocortex
POST	Postsubiculum	Hippocampal Formation
PRE	Presubiculum	Hippocampal Formation
PTLp	Posterior parietal association areas	Isocortex

RSPagl	Retrosplenial area, lateral agranular part	Isocortex
RSPd	Retrosplenial area, dorsal part	Isocortex
RSPv	Retrosplenial area, ventral part	Isocortex
SSp-bfd	Primary somatosensory area, barrel field	Isocortex
SSp-ll	Primary somatosensory area, lower limb	Isocortex
SSp-m	Primary somatosensory area, mouth	Isocortex
SSp-n	Primary somatosensory area, nose	Isocortex
SSp-tr	Primary somatosensory area, trunk	Isocortex
SSp-ul	Primary somatosensory area, upper limb	Isocortex
SSs	Supplemental somatosensory area	Isocortex
SUBd	Subiculum, dorsal part	Hippocampal Formation
SUBv	Subiculum, ventral part	Hippocampal Formation
TEa	Temporal association areas	Isocortex
TR	Postpiriform transition area	Olfactory Areas
TT	Taenia tecta	Olfactory Areas
VISC	Visceral area	Isocortex
VISal	Anterolateral visual area	Isocortex
VISam	Anteromedial visual area	Isocortex
VISl	Lateral visual area	Isocortex
VISp	Primary visual area	Isocortex
VISpl	Posterolateral visual area	Isocortex
VISpm	posteromedial visual area	Isocortex

Sources of sensory cascades

The ten cortical regions we consider as sources of sensory activation cascades are shown in Table SI-2. Their location is shown in Figure SI-2.

Table SI-2: *Sources of sensory cascades*

Acronym	Description	Major region	Size (number of voxels)
AUDp	Primary auditory area	Isocortex	2689
VISp	Primary visual area	Isocortex	6227
GU	Gustatory areas	Isocortex	2104
SSp-n	Primary somatosensory area, nose	Isocortex	1358
SSp-bfd	Primary somatosensory area, barrel field	Isocortex	10306
SSp-ll	Primary somatosensory area, lower limb	Isocortex	3254
SSp-m	Primary somatosensory area, mouth	Isocortex	2924
SSp-tr	Primary somatosensory area, trunk	Isocortex	4799
SSp-ul	Primary somatosensory area, upper limb	Isocortex	5406
MOB	Main olfactory bulb	Olfactory Areas	16978

Sensitivity to activation threshold θ

Recall that if θ is higher than 0.98, the cascade of some sources will not be complete. We have also computed the core nodes for two lower values of θ: 0.9 and 0.95. The 70%-core remains exactly the same. The 90%-core for $\theta = 0.9$ includes PERI instead of ECT.

Similarity between activation cascades with single and complete linkage

Figure SI-3 shows hierarchical clustering dendrograms quantifying the similarity between the ten activation cascades based on single (left) and complete (right) linkage. The corresponding average linkage plot is shown in Figure 3-b.

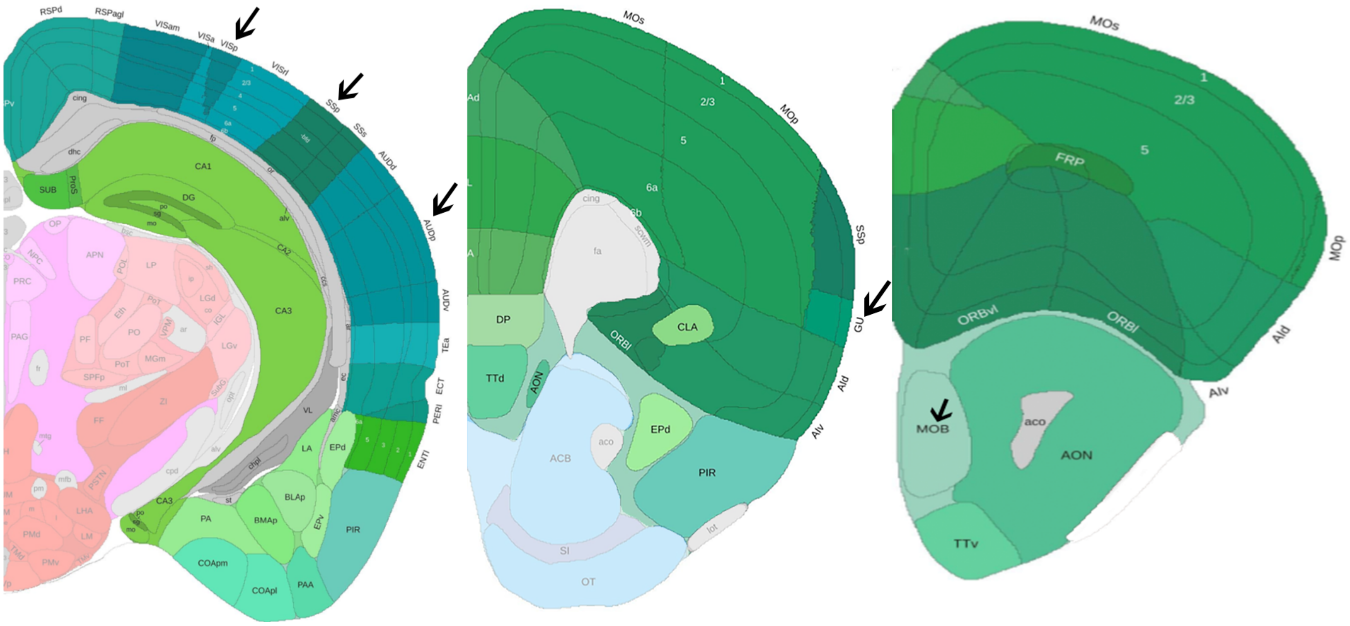

Figure SI-2: *The location of the ten primary sensory regions:* Three coronal slices from the Allen Mouse Brain Atlas with the cerebral cortex regions tinted by green and the source regions identified by an arrow. The somatosensory region includes six different sub-regions for lower limbs, upper limbs, trunk, mouth, nose and whiskers. The remaining four sensory sources are: visual (VISp), auditory (AUDp), gustatory (GU) and olfactory (MOB).

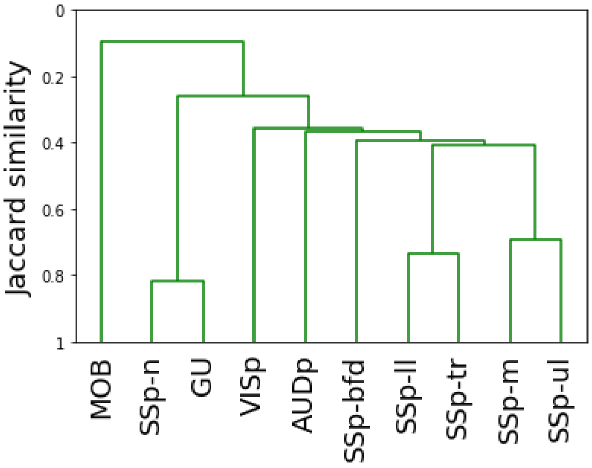

(a) Single linkage

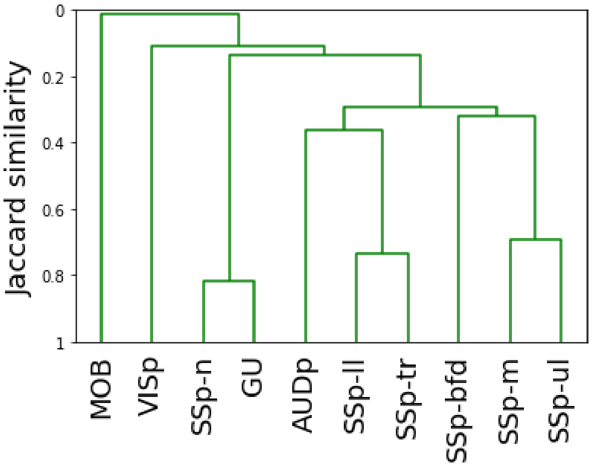

(b) Complete linkage

Figure SI-3: *Similarity between activation cascades.*

Which anatomical connections are more important in sensory cascades?

Which anatomical connections are more important in terms of MSI? Only about half the connections of the anatomical connectome appear in sensory activation cascades, and a quarter of the former appear in only one sensory cascade.

To answer this question, we examine the conditional probability that an connection e_l of physical length l appears in an activation cascade given that $l > l_0$; we denote this probability as $P[e_l = 1|l > l_0]$. Similarly, we define the probability $P[e_w = 1|w > w_0]$ for an edge of weight w.

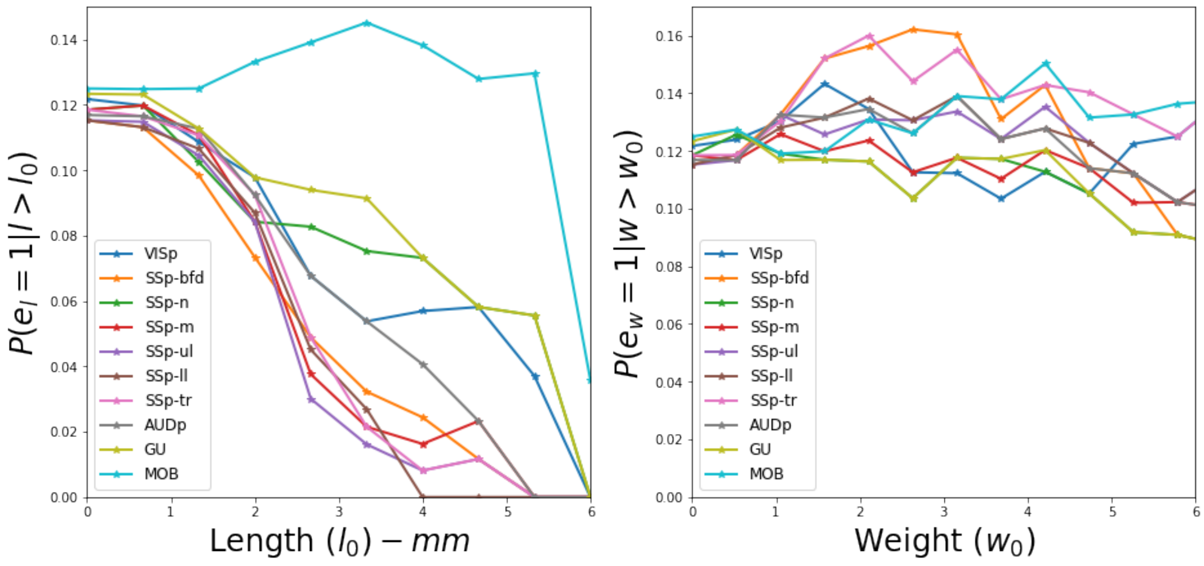

(a) (b)

Figure SI-4: *Connections that appear in sensory cascades:* (a) Conditional probability that a connection e_l of physical length l appears in an activation cascade given that $l > l_0$. (b) Conditional probability that a connection e_w of weight w appears in an activation cascade given that $w > w_0$.

Figure SI-4 shows these two conditional probabilities separately for each of the ten activation cascades. Even though there are significant variations across the ten cascades, all of them show that $P[e_l = 1|l > l_0]$ decreases with l_0, i.e., *as the length of a connection increases (and especially when l is larger than 1-2mm) it becomes less likely that it will be part of an activation cascade.*

The right part of Figure SI-4 shows the corresponding results for the connection weight conditional probability $P[e_w = 1|w > w_0]$. With the exception of the olfactory cascade (MOB), which shows a decreasing trend, the rest of the sensory cascades show a more complex and diverse pattern. The average probability suggests that weight is *not* a significant factor in determining which connections will be part of an activation cascade (perhaps with the exception of edges with very high weights – larger than 8). One reason is that if the weight of a connection is higher than the activation threshold θ (which is about one in our modeling results), that edge is sufficient to activate a downstream node, independent of the state of other connections to the same destination node.

These results suggest that *sensory cascades spread in the cortex as a forest fire mostly through short connections connecting physically adjacent regions, rather than through the (relatively few) long connections that connect remote regions.*

Comparison with other centrality metrics and other network-core notions

We also ask whether the path centrality metric correlates strongly with more commonly used node centrality metrics, namely: incoming or outgoing strength (the equivalent of "degree" for directed and weighted networks), betweenness centrality (fraction of all shortest paths traversing a node), closeness centrality (inversely related to average shortest path distance from that node to any other node), pagerank and eigenvector centrality (two related "influence" metrics that assign a higher score to a node that is connected to other highly-scored nodes compared to a node that has the same number of connections to low-scored nodes) Rubinov and Sporns (2010). Given that we are mostly interested in the dissemination of sensory information over the network, one may expect that the pagerank and eigenvector centrality metrics would be more highly correlated with path centrality because such cascades do not necessarily follow shortest paths Perra and Fortunato (2008).

Centrality metric	Top-five nodes
Out-strength	ENTl - CA2 - PIR - FRP - EPv
In-strength	CA1 - MOs - ENTl - DP - FRP
Betweenness	ENTl - CLA - PERI - VISl - MOs
Closeness	ENTl - PERI - CLA - PIR - VISl
Pagerank	VISl - RSPagl - VISpm - FRP - RSPv
Eigenvector	MOs - FRP - AOB - CLA - MOp
**Path Centrality**	CLA - SSs - PTLp - AUDv - MOs

Table SI-3: Top-five nodes according to different centrality metrics

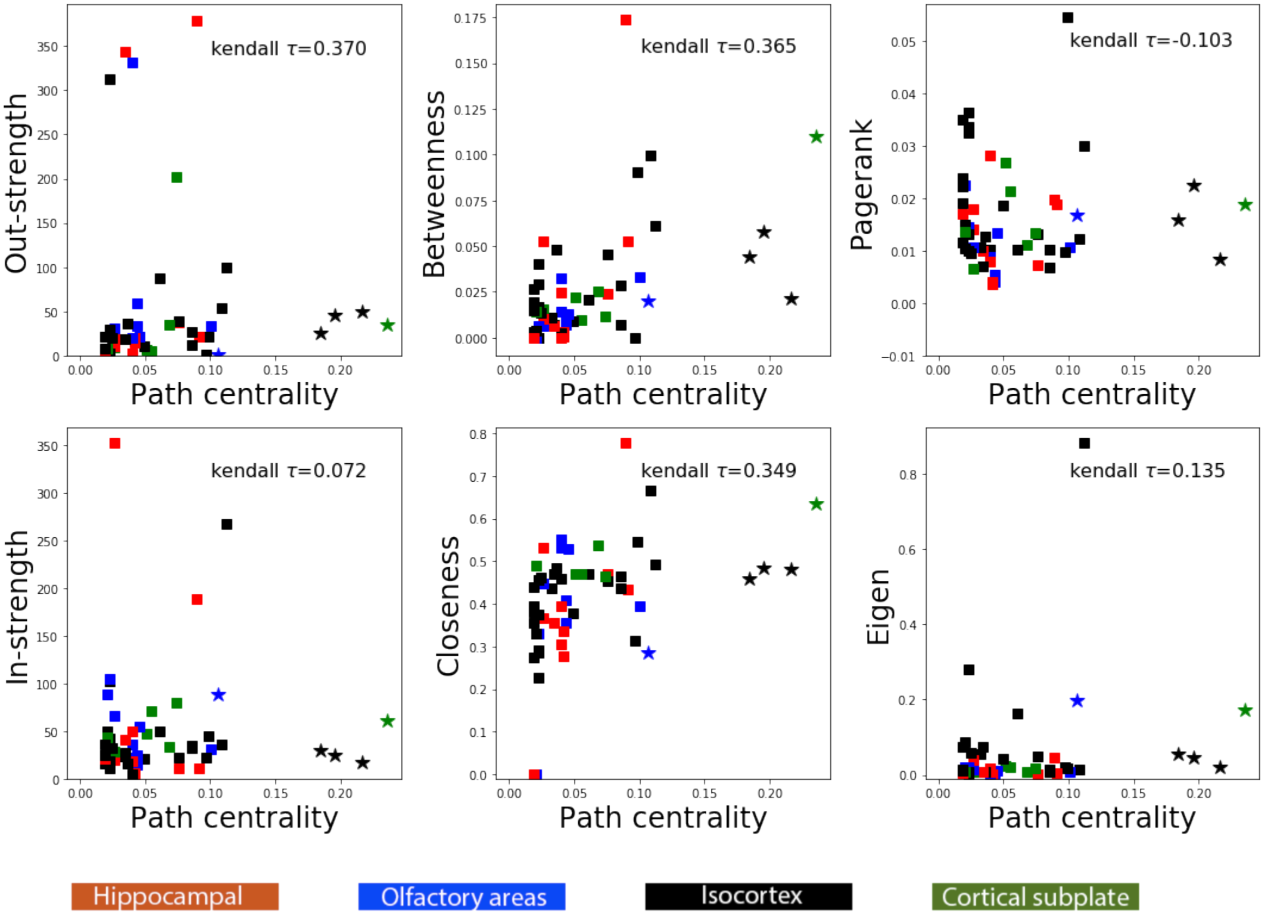

Figure SI-5: *Path centrality compared to other centrality metrics.* Each point corresponds to one of the 67 nodes in the network. The four star nodes constitute the hourglass core for τ=70%. All nodes are color-coded based on the broader brain region they belong to (isocortex, hippocampal formation, cortical subplate, olfactory areas).

Table SI-3 shows the top-five nodes in the network for each centrality metric, while Figure SI-5 shows scatter plots for the previous centrality metrics, comparing each of them with path centrality. The plots also show Kendall's τ rank correlation coefficient. All centrality metrics are computed using ***networkx*** Hagberg, Swart, and S Chult (2008). Strength (either for outgoing or incoming edges) only considers the local connections of each node; nodes in the hippocampal formation (CA1, CA2, ENTl) are among the most strongly connected, while none of the τ-core nodes ranks highly in terms of strength. The betweenness and closeness metrics are both based on shortest paths; the lateral entorhinal (ENTl)

ranks highest in terms of that metric, while the claustrum (CLA) is the only τ-core node in the top-5 according to these two metrics. The highest ranked node based on pagerank is the lateral visual area (VISl) while the highest ranked node based on eigenvector centrality is (by far) the secondary motor area (MOs). In brief, the conclusion of this comparison is that none of these centrality metrics correlates well with path centrality, computed over all source-target sensory activation paths.

Mode	Nodes
Rich club (in order of total degree)	BLA, CLA, ENTl, MOs, PERI
Rombach core (in order of Rombach score Rombach, Porter, Fowler, and Mucha (2017))	CA2, CA1, FRP, ENTl, MOs, EPv, PIR, MOp DP, COAa, NLOT, PAA, AOB
Hourglass core for τ=90% (in order of path coverage)	CLA, PTLp, AUDv, AOB SSs, MOs, ACAd, VISl, ECT

Table SI-4: Rich-club nodes, Rombach core nodes, and hourglass core nodes (τ=90%): the overlap between the first two sets with the hourglass core nodes is highlighted in red.

We also examined the similarity between the nine τ-core nodes, the five rich-club nodes Harriger, Van Den Heuvel, and Sporns (2012); McAuley, da Fontoura Costa, and Caetano (2007); Senden, Deco, de Reus, Goebel, and van den Heuvel (2014), as well as the thirteen core-periphery nodes computed using Rombach's method Rombach et al. (2017). Table SI-4 shows the five rich-club nodes. The rich-club analysis is performed on unweighted and undirected networks and the rich-club coefficient is computed based on 1000 random surrogate networks, as described in McAuley et al. (2007). The rich-club coefficient peaks at 1.15 for nodes with total degree over 23. The overlap between the rich-club and the τ-core nodes consists of only the claustrum (CLA) and the supplementary motor region (MOs).

We also performed a core-periphery analysis on weighted but undirected networks using Rombach's method Rombach et al. (2017). The core nodes according to this method are reported in Table SI-4. Again, only two of the nodes in that core set overlap with the hourglass τ-core (MOs and AOB).

Activation cascades when two sensory sources are activated simultaneously

We have also considered the case of two simultaneously active sources, considering all possible pairs of sources (10x9/2=45 cascades). The hourglass analysis is summarized in Figure SI-6. The core nodes are the same with the single-source case, except that the anterior cingulate area – dorsal part (ACAd) and the Ectorhinal area (ECT) are replaced by the perirhinal area (PERI).

Analysis of "disagreement cases" between VSD data and ALT modeling results

In this section we analyze the cases in which the VSD experimental results predict a different temporal ordering than the ALT modeling results. Recall that a disagreement refers to a pair of ROIs (e.g., X and Y) for which the activation order in ALT is different than that in VSD. Thus, every disagreement case involves two different ROIs.

We first split the ROI pairs in two sets, the disagreement cases and the agreement cases. We measured the Euclidean distance between the center of the two ROIs in each pair of the two sets. The hypothesis that the sample mean of these distances is the same could not be rejected with a p-value of 5%.

We did the same for the connection weight between connected ROI pairs, comparing the average weight of pairs in the set of agreements and the set of disagreements. Again, the hypothesis that the sample mean of these distances is the same could not be rejected with a p-value of 5%.

So, the distance or connection weight between two ROIs does not predict whether they will be a disagreement case.

Are there certain ROIs that appear in surprisingly many disagreement cases? To answer this question, we measure the number of times X that each ROI appears in the N disagreement cases that are observed in the cascade of a certain sensory modality (considering the datasets form all five animals). If an ROI appears in $k > \mu + 3\sigma$ disagreement cases, we conclude that it is significantly over-represented in the group of disagreement cases. The mean and standard deviation in the previous inequality are calculated based on the null model that the disagreements involve randomly chosen pairs of distinct ROIs. So, if K is the number of ROIs and we have N disagreements (for a specific sensory cascade), then the probability of selecting a specific ROI in each pair is:

$$p = \frac{1}{K} + (1 - \frac{1}{K}) \times \frac{1}{K-1} \quad (2)$$

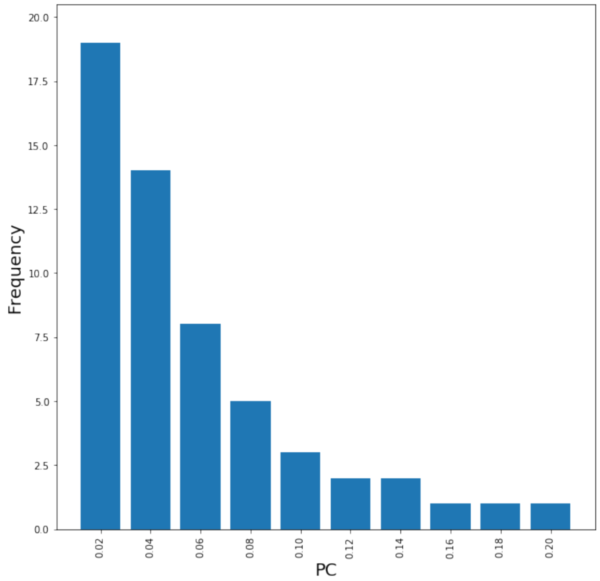

(a)

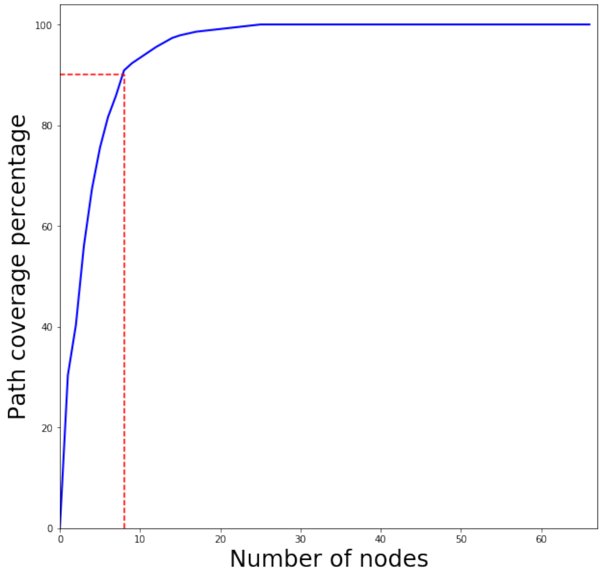

(b)

Node	Description	Path Coverage	PC Rank
CLA	Claustrum	21	1
PTLp	Posterior parietal association areas	18	3
SSs	Supplemental somatosensory area	16	2
AOB	Accessory olfactory bulb	11	6
AUDv	Ventral auditory area	8	5
PERI	Perirhinal area	8	4
MOs	Secondary motor area	5	9
VISl	Lateral visual area	4	11

(c)

Figure SI-6: *Simultaneous activation of two source nodes:* (a) Path Centrality (PC) histogram for the 67 cortical nodes, considering all source-target paths across the 10×9/2=45 activation cascades. (b) Cumulative path coverage by the top-X core nodes for X=$1 \cdots 67$. (c) Eight nodes are enough to cover $\tau = 90\%$ of all paths.

Given that we sample N pairs, the null model is that we will sample each ROI a number of times X, where X follows the $Binomial(N, p)$. So, $\mu = N\,p$ and $\sigma^2 = N\,p\,(1-p)$.

Figure SI-7 shows the results for this study. Note that in each of the five cascades, it is only one or two ROIs that are significantly over-represented in disagreement cases. In the visual cascade for instance, it is the SSp-bfd ROI that is present in surprisingly many disagreements.

We further analyzed these disagreements in which one of the two ROIs is an outlier, asking whether the second ROI is also over-represented. Figure SI-7f identifies such ROI pairs – four of the cascades have only one pair while the Forelimb stimulation cascade has none. ACAd is an ROI that appears in many disagreements in the visual, whisker and tone stimulation cascades – note that this ROI appears at the boundary of the cortical surface at the VSD datasets and it is only partially visible. So, it is likely that the VSD data do not reflect accurately the time at which that region is activated after each stimulation. To a smaller degree, the same may be true for TEa, which is the outlier in the hindlimb stimulation cascade.

Modeling versus experimental results on randomized connectomes

Here, we examine the effect of the four network randomization approaches described in Section on the comparisons between modeling and experimental results. Specifically, we apply the ALT model on the four types of randomized connectomes (randomized weights, lengths, weights and lengths, topology) and compared the resulting cascades with the corresponding VSD-based visual cascade (as in section). The results are shown in Figure SI-8.

As expected based on the analysis of Section , when we compare modeling and experimental results, we observe that topological randomization causes a statistically significant reduction in the percentage of of ”temporal agreement” ROI pairs (Mann-Whitney U-test, $p < 0.05$), in the order of 15-30%. The randomization of lengths and/or weights on the other hand does not have a statistically significant effect on the percentage of ”temporal agreement” ROI pairs.

The ALT cascade of each sensory source

The ten activation cascades, one for each sensory source, are shown in Figures SI-9 through SI-18.

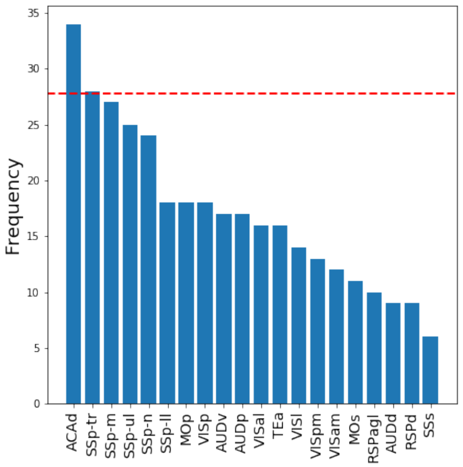

(a) Whisker cascade

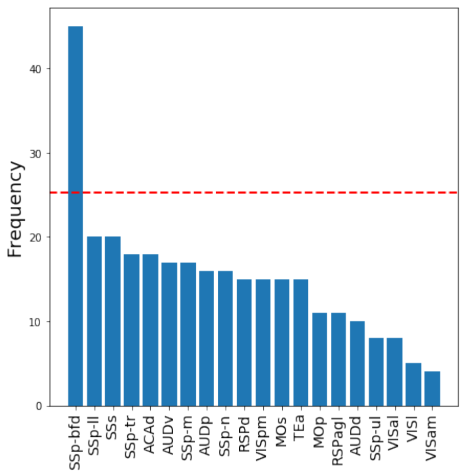

(b) Visual cascade

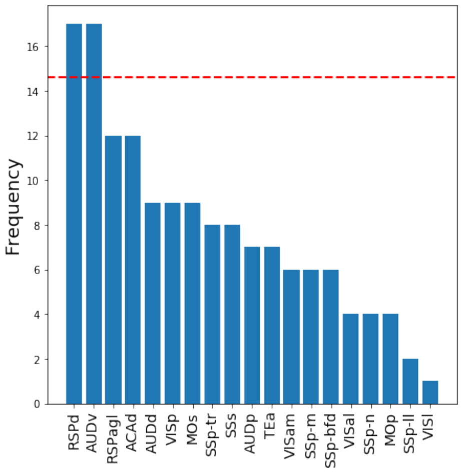

(c) Forelimb cascade

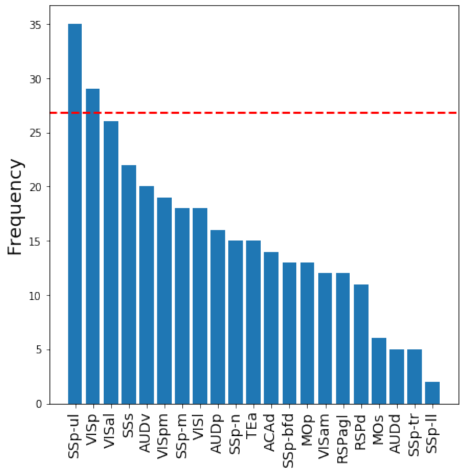

(d) Hindlimb cascade

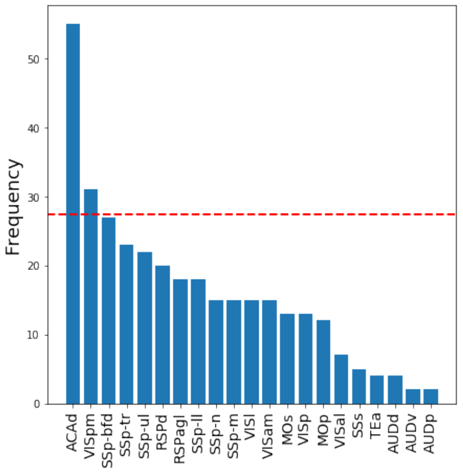

(e) Tone cascade

Stimulus	First ROI	Second ROI
Visual	ACAd*	SSp-bfd
Whisker	ACAd*	AUDp
Tone	ACAd*	SSp-ul
Hindlimb	TEa*	VISp

(f) Disagreement ROI pairs

Figure SI-7: (a)-(e): The number of disagreements between VSD and ALT that involve each ROI, over the 21 ROIs that appear in the VSD data (Continued on the following page).

Figure SI-7: The outlier ROIs have frequencies that exceed the red dashed line. (f) Disagreement ROI pairs that appear in all five animal datasets - each row corresponds to a different stimulus. ROIs at the boundary of the VSD visible cortical surface are marked by a star.

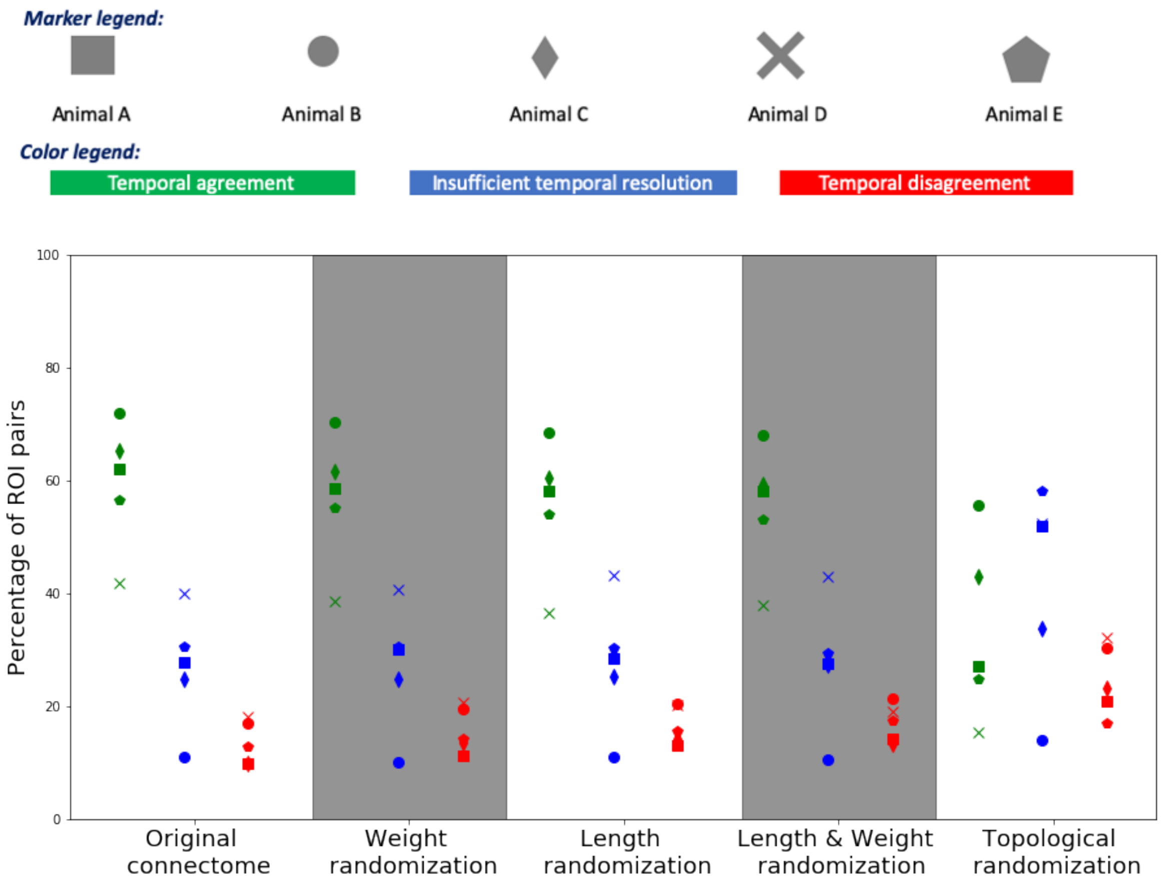

Figure SI-8: *Comparison between model-based and experimental temporal ordering of ROI activations for the visual cascade in randomized connectomes:* a) The y-axis shows the percentage of (X, Y) ROI pairs that show temporal agreement (green), temporal disagreement (red), and insufficient temporal resolution (blue) between the activation order of X and Y in the modeling results and the mouse experiments. The plot shows results for five animals, and for the four types of randomized connectomes described in Section .

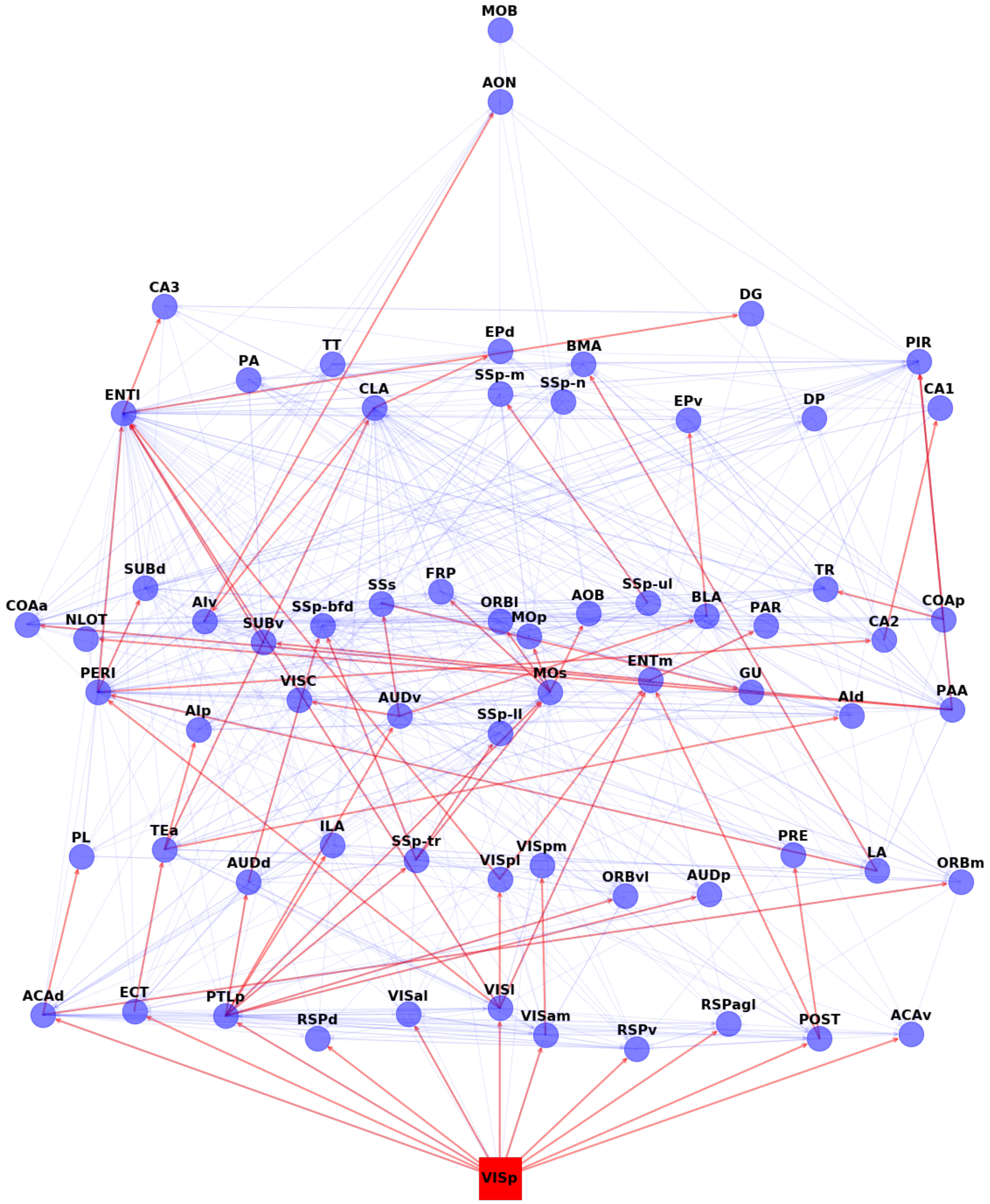

Figure SI-9: *Visual cascade* (source: VISp). Also see Figure 4 for additional details about this visualization.

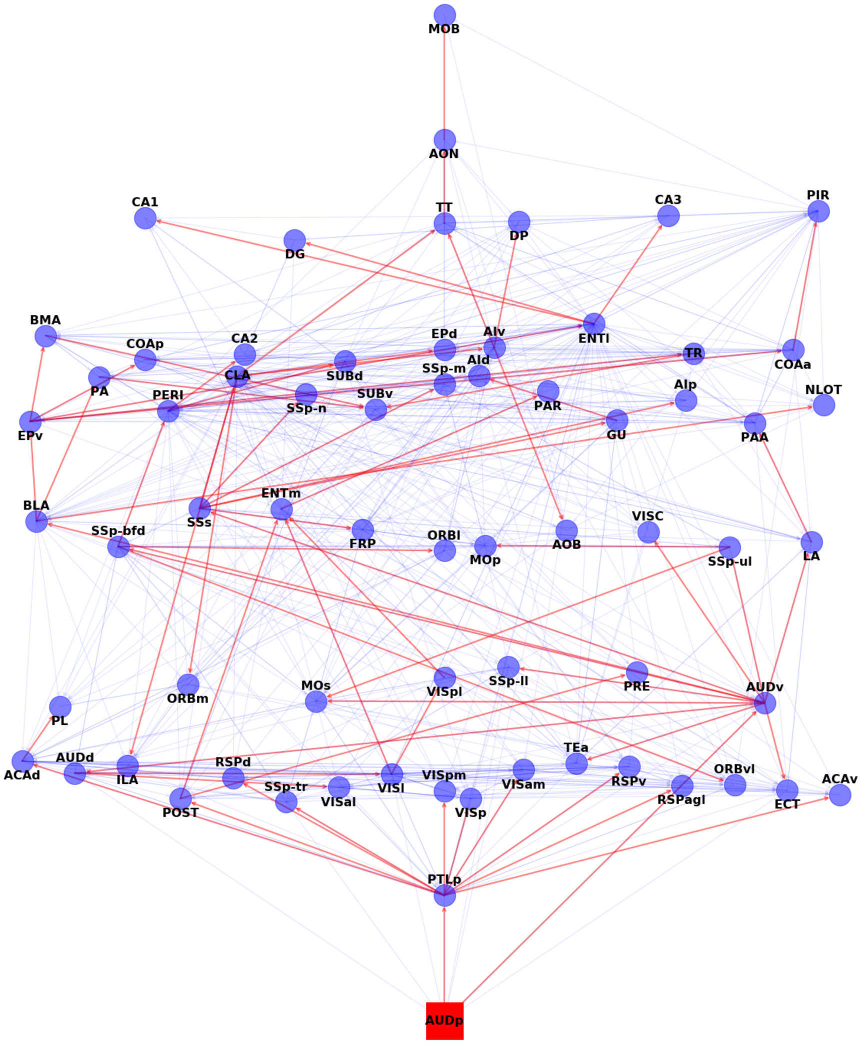

Figure SI-10: *Auditory cascade* (source: AUDp). Also see Figure 4 for additional details about this visualization.

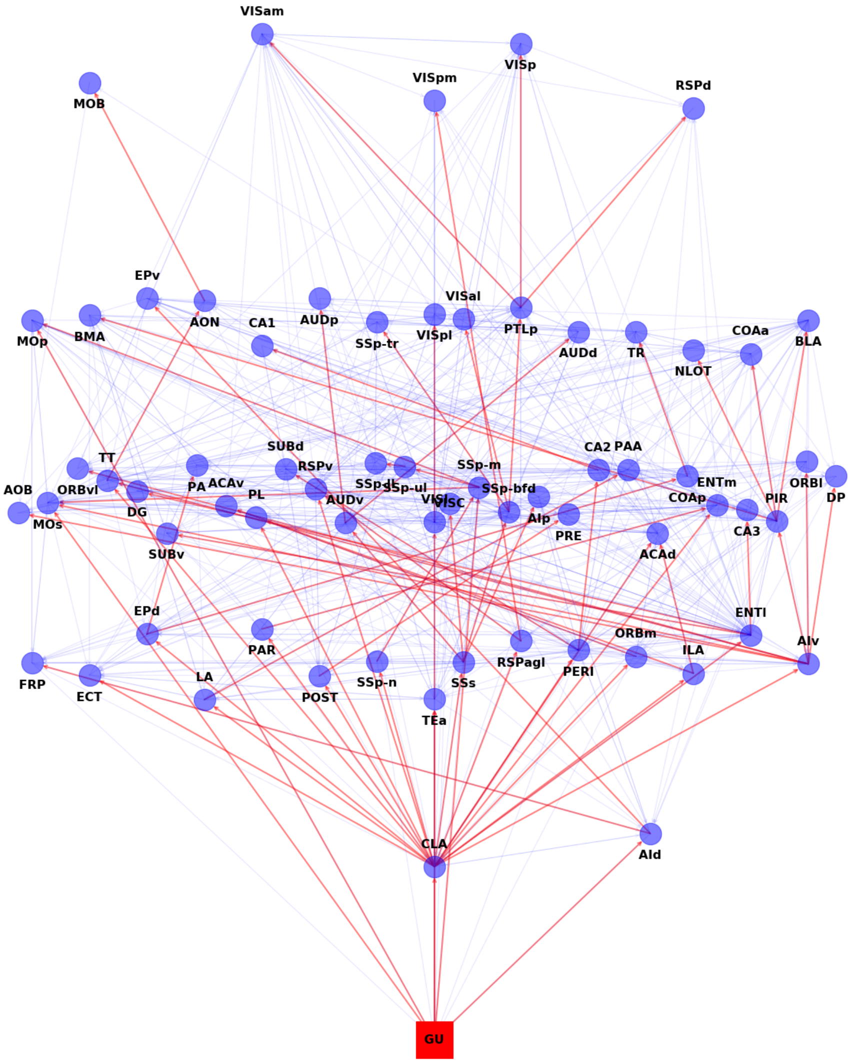

Figure SI-11: *Gustatory cascade* (source: GU). Also see Figure 4 for additional details about this visualization.

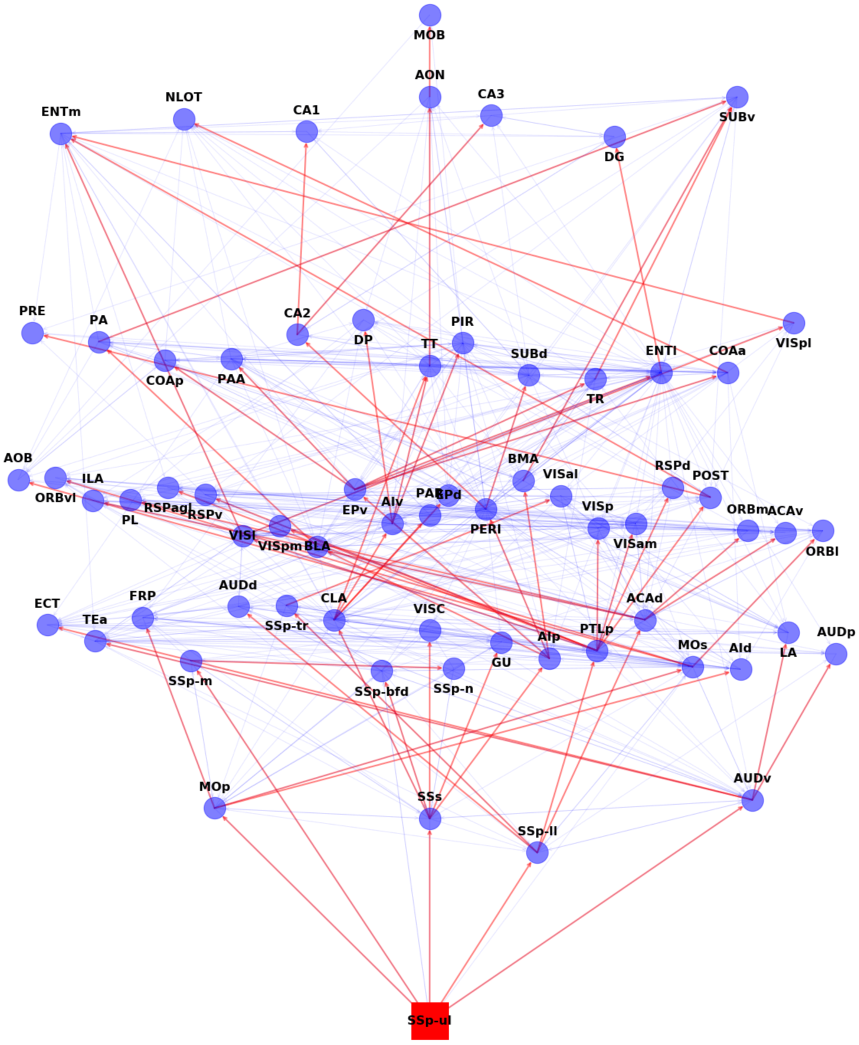

Figure SI-12: *Upper-limb somatosensory cascade* (source: SSp-ul). Also see Figure 4 for additional details about this visualization.

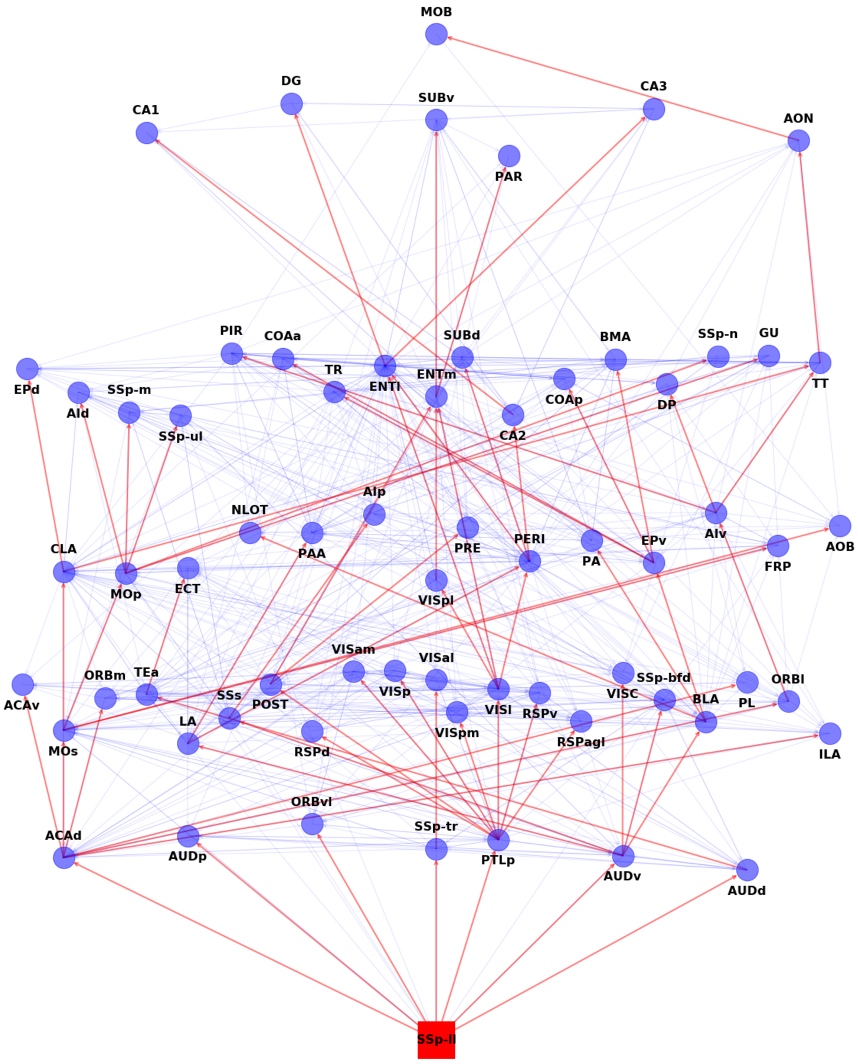

Figure SI-13: *Lower-limb somatosensory cascade* (source: SSp-ll). Also see Figure 4 for additional details about this visualization.

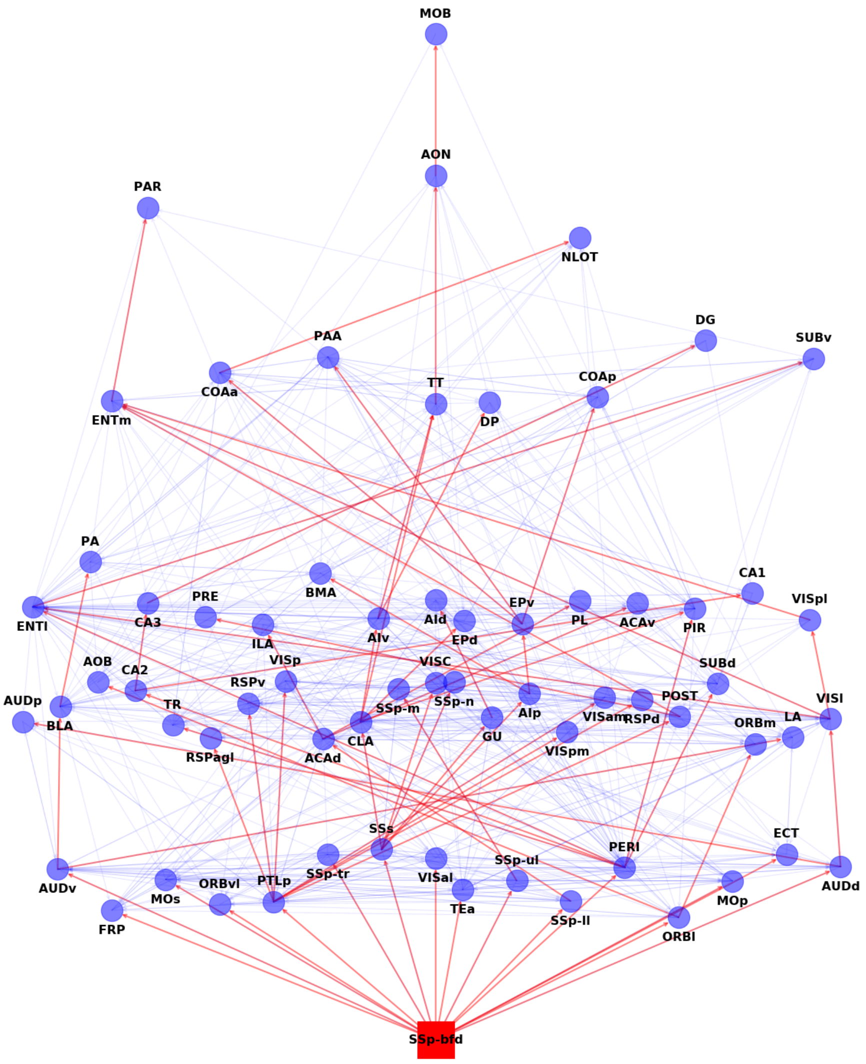

Figure SI-14: *Whiskers somatosensory cascade* (source: SSp-bfd). Also see Figure 4 for additional details about this visualization.

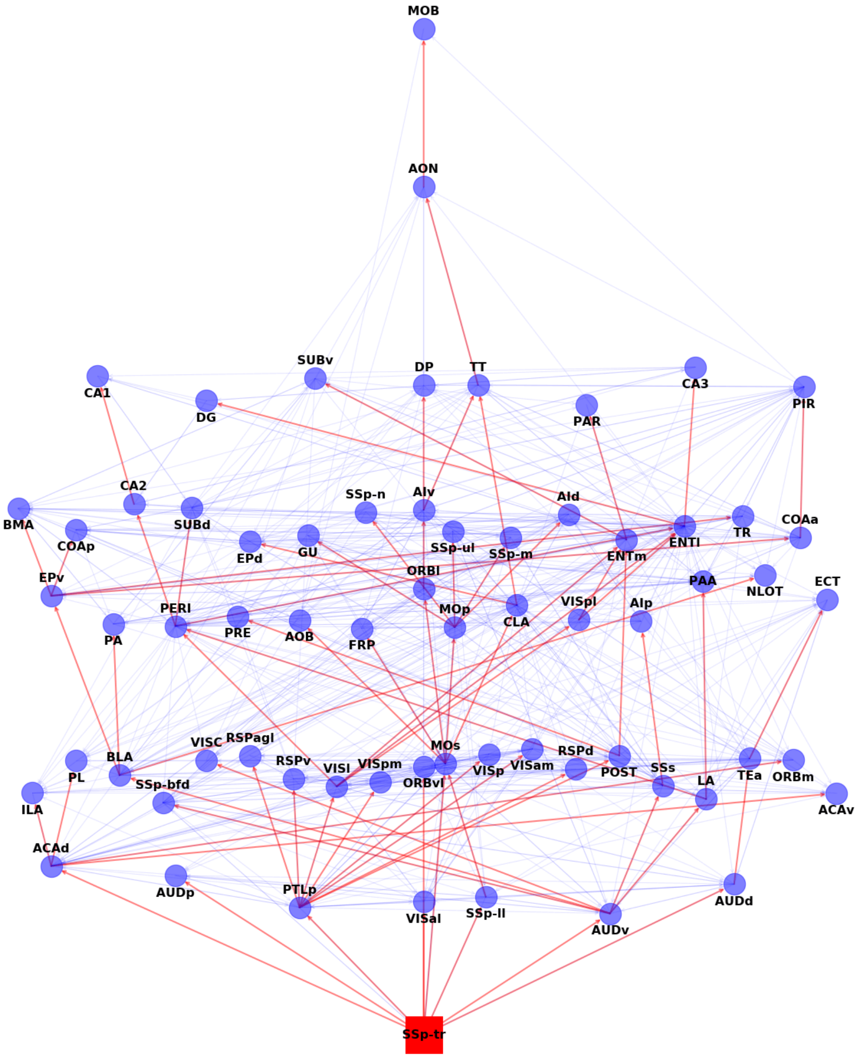

Figure SI-15: *Trunk somatosensory cascade* (source: SSp-tr). Also see Figure 4 for additional details about this visualization.

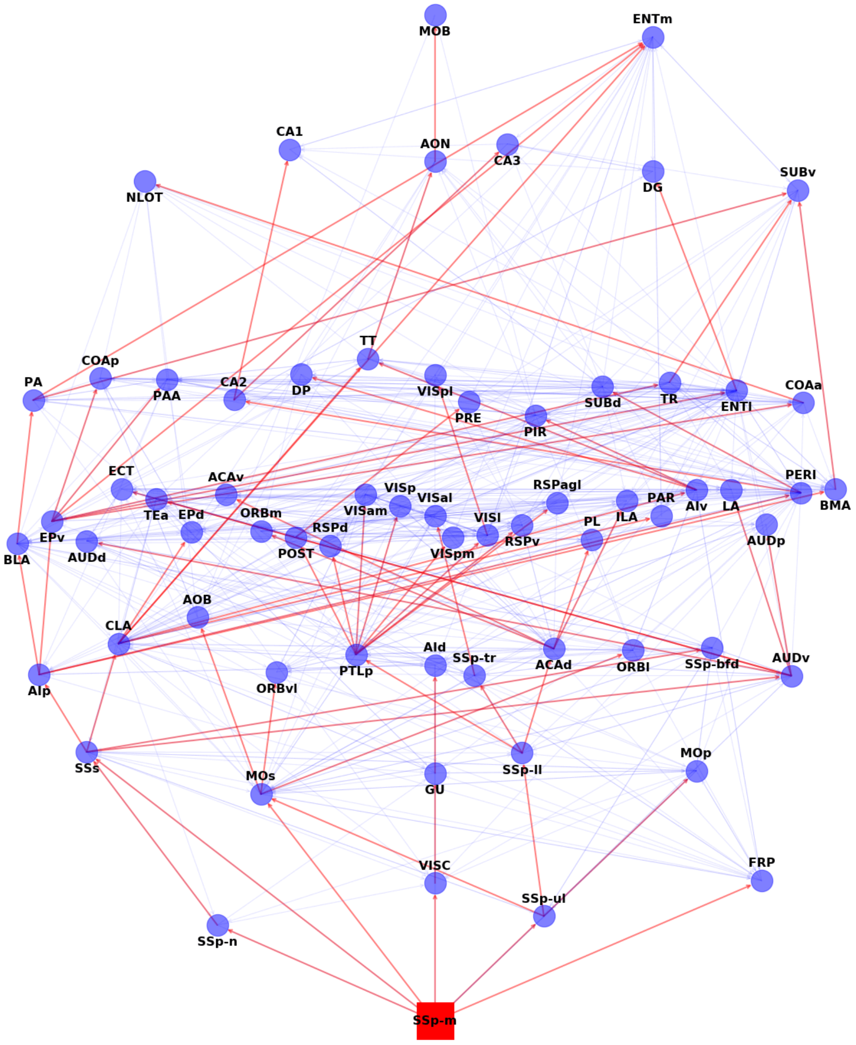

Figure SI-16: *Mouth somatosensory cascade* (source: SSp-m). Also see Figure 4 for additional details about this visualization.

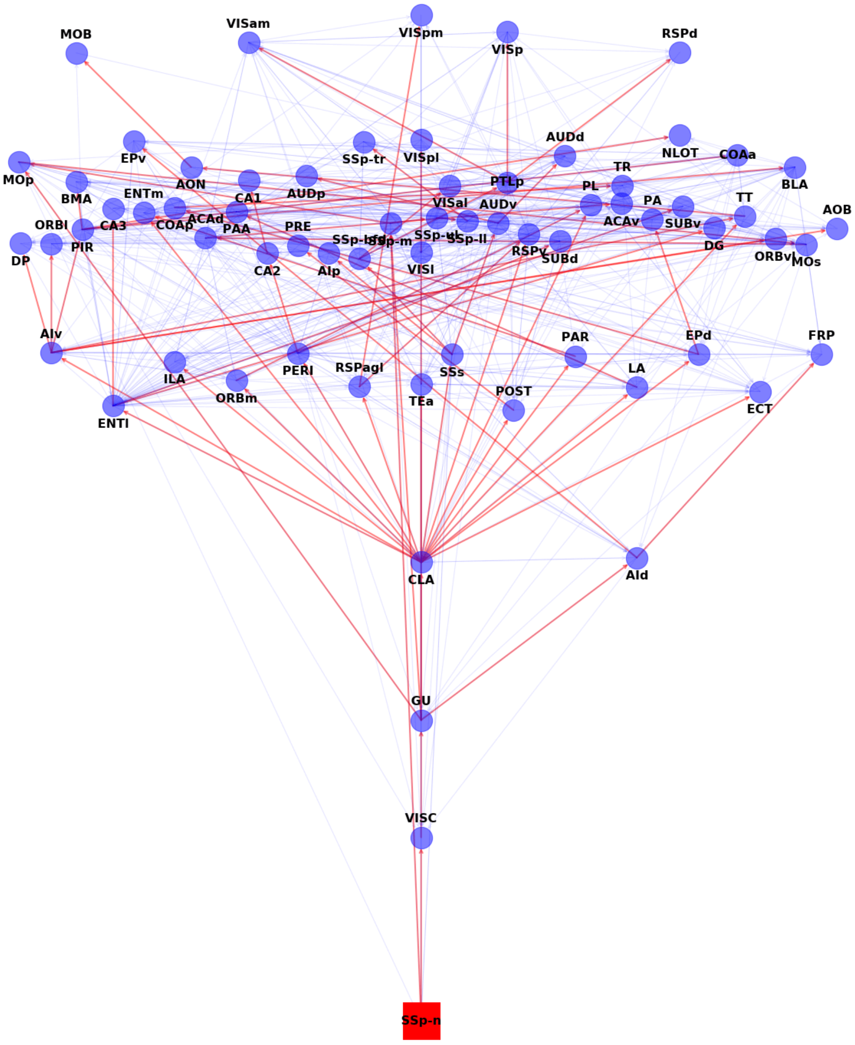

Figure SI-17: *Nose somatosensory cascade* (source:SSp-n). Also see Figure 4 for additional details about this visualization.

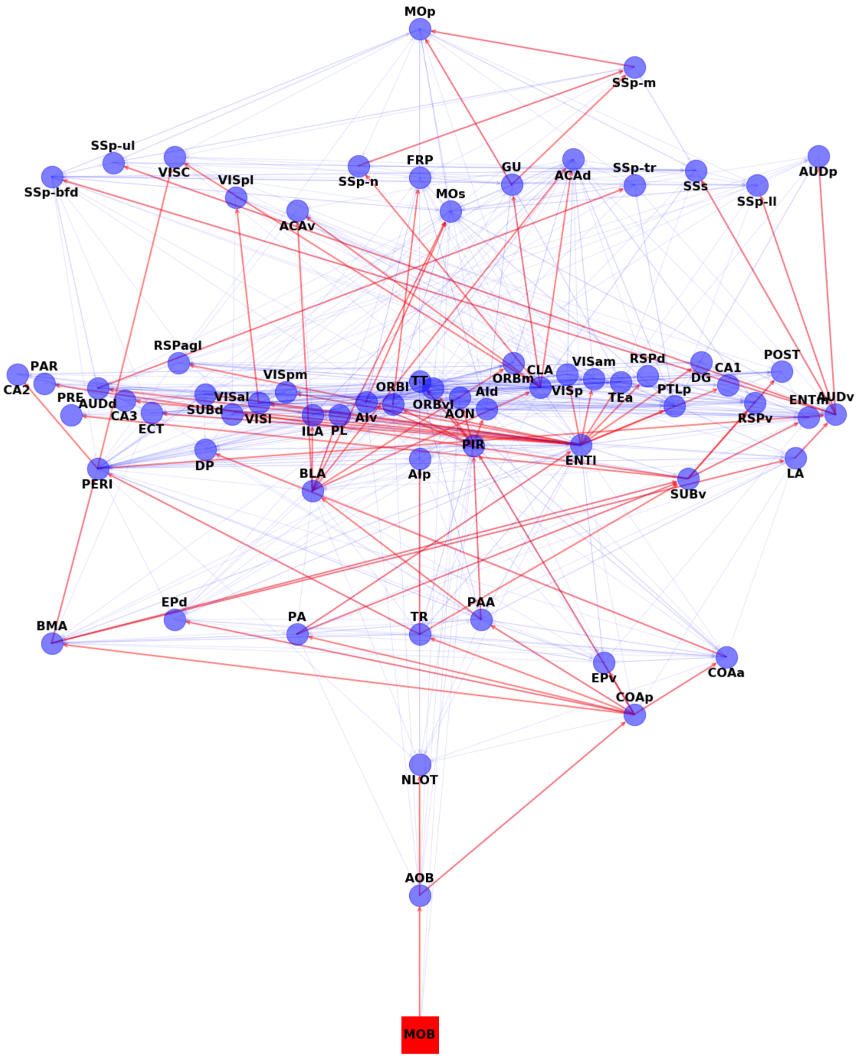

Figure SI-18: *Olfactory cascade* (source: MOB). Also see Figure 4 for additional details about this visualization.
